# Supplementary material for: Barriers and facilitators to pressure ulcer prevention behaviours by older people living in their own homes and their lay carers: a qualitative study
Source: BMJ Open. 2024 Mar 18;14(3):e080398. doi: 10.1136/bmjopen-2023-080398 (PMC10953097; doi:10.1136/bmjopen-2023-080398)
Supplement: Supplementary data [file bmjopen-2023-080398supp001.pdf]

Supplementary Table 1: Community health and social services supporting patients (✔) and carers (*) in the last year

Pseudonym	Role	Carer centre	Day centre	District nurse	Family carer live-in	Family carer visiting	GP	Occupational therapist	Paid carer live-in	Paid carer visiting	Pharmacy	Physiotherapist	Podiatrist	Residential respite	Sitter	Social prescriber	Social worker	Specialist nurse	Speech and language	Tissue viability nurse
Pt1	Patient			✔		✔		✔	✔	✔										
Pt2	Patient			✔		✔		✔			✔	✔								
Pt3	Patient			✔		✔	✔	✔	✔	✔										
Pt4	Patient			✔								✔								
Pt5	Patient			✔	✔															
Pt6	Patient			✔	✔		✔	✔												
Pt7	Patient			✔	✔															
Pt8	Patient			✔		✔	✔	✔		✔	✔	✔	✔			✔				
Pt9	Patient		✔	✔	✔	✔				✔										✔
Pt10	Patient			✔	✔	✔	✔				✔	✔		✔						
Ca1	Carer			✔	✔	✔	✔	✔		✔		✔	✔		*					
Ca2	Carer	*		✔	✔	✔	✔					✔	✔							✔
Ca3	Carer	*			✔			✔		✔			✔							
Ca4	Carer			✔	✔							✔								
Ca5	Carer			✔	✔		✔	✔												
Ca6	Carer				✔	✔	✔				✔	✔		*						
Ca7	Carer			✔	✔	✔		✔		✔				*			✔			
Ca8	Carer	*		✔	✔		✔	✔							✔		✔			
Ca9	Carer			✔		✔	✔	✔		✔					✔			✔	✔	
Ca10	Carer			✔		✔		✔		✔		✔		*						
