# Supplementary material for: Barriers and facilitators to pressure ulcer prevention behaviours by older people living in their own homes and their lay carers: a qualitative study
Source: BMJ Open. 2024 Mar 18;14(3):e080398. doi: 10.1136/bmjopen-2023-080398 (PMC10953097; doi:10.1136/bmjopen-2023-080398)
Supplement: Author's manuscript [file bmjopen-2023-080398.draft_revisions.pdf]

BMJ Open is committed to open peer review. As part of this commitment we make the peer review history of every article we publish publicly available.

When an article is published we post the peer reviewers' comments and the authors' responses online. We also post the versions of the paper that were used during peer review. These are the versions that the peer review comments apply to.

The versions of the paper that follow are the versions that were submitted during the peer review process. They are not the versions of record or the final published versions. They should not be cited or distributed as the published version of this manuscript.

BMJ Open is an open access journal and the full, final, typeset and author-corrected version of record of the manuscript is available on our site with no access controls, subscription charges or pay-per-view fees (http://bmjopen.bmj.com).

If you have any questions on BMJ Open's open peer review process please email info.bmjopen@bmj.com

BMJ Open

Barriers and facilitators to pressure ulcer prevention behaviours by older people and their lay carers in community settings

Journal:	*BMJ Open*
Manuscript ID	bmjopen-2023-080398
Article Type:	Original research
Date Submitted by the Author:	29-Sep-2023

Complete List of Authors:	Roddis, Jennifer; University of Portsmouth, School of Health and Care Professions<br>Dyson, Judith; Birmingham City University, C-SCHaRR<br>Woodhouse, Marjolein; University of Portsmouth, School of Health and Care Professions<br>Devrell, Anne; Patient and Public Involvement representative<br>Oakley, Karen; Solent NHS Trust<br>Cowdell, Fiona; Birmingham City University, Faculty of Health Education and Life Sciences

Keywords:	Aging, Nursing Care, Patients, Primary Prevention, QUALITATIVE RESEARCH

SCHOLARONE™ Manuscripts

I, the Submitting Author has the right to grant and does grant on behalf of all authors of the Work (as defined in the below author licence), an exclusive licence and/or a non-exclusive licence for contributions from authors who are: i) UK Crown employees; ii) where BMJ has agreed a CC-BY licence shall apply, and/or iii) in accordance with the terms applicable for US Federal Government officers or employees acting as part of their official duties; on a worldwide, perpetual, irrevocable, royalty-free basis to BMJ Publishing Group Ltd ("BMJ") its licensees and where the relevant Journal is co-owned by BMJ to the co-owners of the Journal, to publish the Work in this journal and any other BMJ products and to exploit all rights, as set out in our licence.

The Submitting Author accepts and understands that any supply made under these terms is made by BMJ to the Submitting Author unless you are acting as an employee on behalf of your employer or a postgraduate student of an affiliated institution which is paying any applicable article publishing charge ("APC") for Open Access articles. Where the Submitting Author wishes to make the Work available on an Open Access basis (and intends to pay the relevant APC), the terms of reuse of such Open Access shall be governed by a Creative Commons licence – details of these licences and which Creative Commons licence will apply to this Work are set out in our licence referred to above.

Other than as permitted in any relevant BMJ Author's Self Archiving Policies, I confirm this Work has not been accepted for publication elsewhere, is not being considered for publication elsewhere and does not duplicate material already published. I confirm all authors consent to publication of this Work and authorise the granting of this licence.

For peer review only

Barriers and facilitators to pressure ulcer prevention behaviours by older people and their lay carers in community settings

Objective: To identify barriers and facilitators to pressure ulcer (PU) prevention behaviours in community dwelling older people and their lay carers.

Design: Theoretically informed qualitative interviews with two phase, deductive then inductive thematic analysis.

Setting: The study was conducted in one geographical region in the United Kingdom, spanning several community NHS Trusts.

Participants: Community dwelling older patients at risk of pressure ulcer development (n=10) and their lay carers (n=10).

Results: Six themes and subthemes were identified: i) knowledge and beliefs about consequences (nature, source, timing and taboo), ii) social and professional role and influences (who does what, conflicting advice and disagreements), iii) motivation and priorities (competing self-care needs and carer physical ability), iv) memory, v) emotion (carer exhaustion and isolation, carer role versus partner role and patient feelings) and vi) environment (human resource and response, and equipment).

Conclusions: There is minimal research in pressure ulcer prevention in community dwelling older people. This study has robustly applied the theoretical domains framework to understanding barriers and facilitators to pressure ulcer prevention behaviours. Our findings will support co-design of strategies to promote preventative behaviours and are likely to be transferable to comparable health care systems nationally and internationally.

Strengths and limitations

- A structured theoretical approach was adopted to data collection and analysis
- Service users were involved in the design, analysis and reporting of this research
- Unlike previous hospital-based research, this study considers community-dwelling patients living in their own homes, and their lay carers
- Recruitment was undertaken in a single geographical area. Whilst our results may be transferable, it is possible that elements of service provision may differ both nationally and internationally.
- The project led to a limited number of facilitators of pressure ulcer prevention behaviours being identified.

For peer review only - http://bmjopen.bmj.com/site/about/guidelines.xhtml

Barriers and facilitators to pressure ulcer prevention behaviours by older people and their lay carers in community settings

Introduction

Pressure ulcers (PUs) are localised injuries to the skin and underlying tissues, usually occurring over a bony prominence, resulting from pressure, or pressure combined with shear[1]. Globally, a PU prevalence of 12.8% and 11.6% has been reported among hospitalised patients[2] and nursing home residents[3], respectively. Pressure ulcers lead to an increased risk of hospital admission, and treatment is costly, placing a substantial burden on care providers[4]. PUs have a detrimental impact on the quality of life of affected individuals[5 6] and are associated with increased morbidity, mortality, pain, fear and despondency[6 7]. Whilst international guidelines recommend PU prevention behaviours, these focus on a hospital population and there is a dearth of research surrounding transferability to community settings[8]. A Priority Setting Partnership involving 500 patients, lay carers and healthcare practitioners (HCP) identified *Understanding the impact of patient and lay carer involvement* as the second most important priority for PU prevention[9].

Existing evidence-based guidelines offer staff intensive PU prevention strategies including i) repositioning at least every four to six hours, ii) use of appropriate devices (e.g., pressure-relieving mattresses), iii) frequent skin inspection and skin care, and iv) optimal nutrition and hydration[10 11]. Although these strategies are appropriate in all settings, home care has unique challenges that limit their applicability. Nursing staff and paid carers are able to spend only limited time with patients, so engagement of patients and lay carers in PU prevention is essential. However, there is limited empirical evidence regarding adherence of patients and lay carers to PU prevention guidance. To date, interventions to enhance patient and lay carer PU prevention behaviours have focused on education, though these have had uncertain impact on knowledge or the number of new pressure ulcers[11]. This suggests knowledge might not be the only factor for non-adherence[12].

When developing strategies or interventions to support health behaviour change, explicit use of theory allows an understanding of behavioural determinants (barriers and facilitators), thus enabling mapping of empirically tested behaviour change techniques[13 14]. The Theoretical Domains Framework (TDF)[15] synthesises all published theories of behaviour or behaviour change into 11 accessible theoretical domains. These domains offer a comprehensive framework of all potential determinants of behaviour (*knowledge, skills, social/professional role and identity, beliefs about capabilities, beliefs about consequences, motivation and goals, memory attention and decision processes, environmental context and resources, social influences, emotion and action planning*).

For peer review only - http://bmjopen.bmj.com/site/about/guidelines.xhtml

Identifying key determinants using the TDF supports identification of the behaviour change techniques (BCTs) most likely to be effective[16].

Objective

To identify barriers and facilitators to PU prevention behaviours in community dwelling older people and their lay carers.

Methods

Design

Qualitative interviews were conducted to elicit barriers and facilitators to PU prevention behaviours. The interview schedule was informed by domains of the TDF.

Participants

Participants were older adults and lay carers. Older adults were community dwelling in their own home, aged ≥65 years, receiving community healthcare and assessed as being at risk of developing a PU (irrespective of current and previous PU status). Lay carers were those providing unpaid care (for people fitting our criteria for older adult participation), of any kind (e.g., physical, household or other practical support). We aimed to recruit a minimum of ten patients and ten carers (n=20) which we expected to be sufficient to achieve data saturation given the theoretical underpinning of the study[17].

Patient and public involvement

In this study we had the input of two patient and public involvement colleagues. One contributed to the study design and the other to design, interpretation of data, reading and commenting on results and writing up. We worked closely with the local Carers Centre who advised on and supported recruitment and hosted a dissemination event for the local community.

Recruitment

Patient participants were recruited through HCPs at a community NHS Trust who provided written information. Several patients declined to take part as they felt that the study was not relevant to their circumstances. Carers were recruited through leaflets distributed through a range of outreach events and community settings, for example, carers centre, churches, community centres, voluntary organisations and supermarkets. Snowballing techniques were used to enhance recruitment. Potential participants were provided with information about the project, including the reasons for the study. Interested individuals were contacted by the research team to discuss the study and decide if they wished to participate.

For peer review only - http://bmjopen.bmj.com/site/about/guidelines.xhtml

Data collection

Consent was gained at the start of the interview. Interviews took place in a venue of the participants choice (usually their own home). Interviews were undertaken by female, post-doctoral researchers (CS, MW or JR) who were not known to participants prior to being recruited into the research, and who introduced themselves and their backgrounds at the start of the interviews. Participants were offered a gift voucher or BACS payment post-interview. Single interviews were audio recorded and transcribed verbatim. Throughout most interviews, only the researcher and participant were present, though on occasion the person cared for, or the carer were present. One interview was undertaken jointly with the patient and their carer. Notes were taken by the researcher as prompts, these did not form part of the analysis. Transcripts were not returned to participants for comment, but feedback on the findings was sought from our PPI representative.

Data analysis

Transcripts were imported into NVivo (v12) and underwent a two-stage analysis. After deductively categorising data to the domains of the TDF, inductive thematic analysis was undertaken according to a six-step process (familiarisation, code generation, combining codes to themes, reviewing themes, determining theme significance and reporting)[18]. Data analysis and collection were iterative processes and continued until saturation was achieved. Analysis was initially undertaken by JD, MW and JR and reviewed by the author team.

Results

Characteristics of participants

Table 1 presents participant characteristics (n=20). Patient participants (women n=4, men n=6) were aged 68 to 94 years (mean age 79.5). Three had a current PU, four had previously had PU(s), two had never had a PU and one currently had and had previously experienced PUs. Of those that had ever had a PU, five had developed these in hospital and three at home. All patient participants had limited mobility. Lay carer participants (women n=7, men n=3) supported or had supported older relatives, and all but two of these relatives had PUs. Ages ranged from 56 to 82 years (mean 75.0). Eight lay carers were caring for their partner (husband or wife), one was caring for their mother-in-law and father-in-law, and one for their mother.

For peer review only - http://bmjopen.bmj.com/site/about/guidelines.xhtml

Table 1: Participant Characteristics[1]

Pseudonym	Role	Gender	Age	Occupation	Health and mobility status of patient	Health status of lay carer	Patient PU status
George	Patient	Male	68	Retired teacher	Lower body paralysis, wheelchair to mobilise		Previous hospital acquired sacral PU
Joni	Patient	Female	94	Retired shop worker	Multiple long-term conditions (LTCs), poor mobility, mobilises using a walking frame		Previous hospital acquired sacral PU
Paul	Patient	Male	72	Retired car manufacturer	Paralysis of legs, wheelchair to mobilise		Current hospital acquired sacral PU
Mick	Patient	Male	81	Retired from the armed forces	Usually mobile, currently compromised mobility post-surgery		Current hospital acquired sacral PU
Carly	Patient	Female	67	Retired teacher	Limited mobility using aids (walking frame, stairlift, rails, mobility scooter)		Previous home acquired sacral and foot PUs
John[1]	Patient	Male	92	Retired engineer	Multiple LTCs, usually mobile with a recent period of immobility		Previous home acquired sacral PU
Tina[2]	Patient	Female	74	Retired secretary	Walks short distances and uses a wheelchair		Nil
Bob	Patient	Male	90	Retired joiner	Multiple LTCs, walks at home with frame and two people, otherwise wheelchair user		Nil
Marvin[3]	Patient	Male	72	Retired police officer	Paraplegic, wheelchair user		Current home acquired sacral PU and previous

[1] Where patient and carer were related this is indicated by superscript matching numbers next to participant pseudonym.

For peer review only - http://bmjopen.bmj.com/site/about/guidelines.xhtml

Pseudonym	Role	Gender	Age	Occupation	Health and mobility status of patient	Health status of lay carer	Patient PU status
							hospital acquired sacral and hip PUs
Linda[4]	Patient	Female	75	Childcare/ nursery	Multiple LTCs, wheelchair user		Current home acquired sacral PU
Cass	Wife caring for husband (now deceased)	Female	81	Retired administrator	Immobile	Osteoporosis	Current home and hospital acquired sacral PUs
Janis[3]	Wife caring for husband	Female	73	Retired administrator	Multiple LTCs and progressive paralysis	Good physical health	Current home acquired sacral PU and previous hospital acquired sacral and hip Pus
Nina	Wife caring for husband	Female	82	Retired administrator	Multiple LTCs, mobile with frame and support, uses wheelchair outdoors	Good physical health	Nil
Jimmy[2]	Husband caring for wife	Male	74	Retired mechanical engineer	Multiple LTCs, limited mobility	Good physical health	Nil
Patsy[1]	Wife caring for husband	Female	81	Retired Nurse	Multiple LTCs, limited mobility	Severe arthritis	Previous hospital acquired sacral PU
Frank[4]	Husband caring for wife	Male	78	Retired Educator	Multiple LTCs, limited mobility	Good physical health	Current home acquired sacral PU
Martha	Daughter-in-law caring for mother and father-in-law (now in care home)	Female	56	Dentist	Both have multiple LTCs and mobilise indoors only with walking frames	Good physical health	1. Home acquired PU lower back/spine 2. nil.
Glen	Son caring for mother	Male	69	Retired surveyor	Limited mobility indoors	Good physical health	Current hospital acquired foot PU
Diana	Wife caring for husband	Female	82	Retired Civil Servant	Multiple LTCs, limited mobility with frame	Arthritis	Previous home acquired PU (site NR)
Patti	Wife caring for husband	Female	74	Retired Teacher	Paralysis, usually wheelchair bound, currently bedbound.	Good physical health	Current hospital acquired multiple site PUs

For peer review only - http://bmjopen.bmj.com/site/about/guidelines.xhtml

Findings

Interviews took a mean of 50 minutes. Six themes (domains of the TDF) and 10 sub-themes were identified (figure 1), each of which is reported below. There was little difference between the barriers and facilitators identified by carers when compared to those identified by patients, but where differences existed, they too are identified below.

Figure 1

Theme 1: Knowledge and beliefs about consequences

Participants generally knew about the strategies to prevent PUs, except for good hydration and nutrition. This knowledge was generated from multiple sources however, in most cases was acquired *after* PU development. The taboo nature of the subject hindered *pre*-PU awareness.

Nature of knowledge

Some found the term pressure ulcer unfamiliar, for example *"it's naivety in that I thought pressure sores were bed sores, so, I thought you only got them in bed"* (Marvin, patient). The three PU prevention strategies known to participants included repositioning and risk of friction when moving, for example, Patsy (carer) stated, *"caused by people spending too much time in one position . . . it limits the blood supply to the skin in that area"*. Many were aware of vulnerable areas particularly referring to the "bottom" with some mentioning back, hips, heels and elbows. Devices were mentioned by most and many used aids to enhance mobility such as frames and grab rails. Patient and lay carer participants reported inspecting at-risk areas of the skin and raising concerns with HCPs. Although some participants appreciated the value of good hydration and nutrition, this was rarely linked to prevention of PUs.

Source, timing and taboo

The main source of knowledge for participants was HCPs (in hospital or at home), sometimes because they gave advice but often because the participant observed what they did and adopted these behaviours. Pete (patient) told us *"I've never received any information . . . it's really information I overhear from things like nurses . . . I've looked it up on the internet . . . I've learnt how to protect myself."* Some participants had received leaflets from HCPs but found them of limited value, for example, *"if you get a wad of paper the feeling immediately is to dismiss it"* (George, patient). Other knowledge sources included Carers' Centre courses and support groups. The words

For peer review only - http://bmjopen.bmj.com/site/about/guidelines.xhtml

of Cass (carer) captured the general consensus *"there definitely needs to be more information out there"* and many highlighted their lack of ability to access online information.

Participants learned about PUs *after* they or their relative had developed one. For example, *"the ward did initially* [told me about PUs], *but it was so big . . . said look, this is what we call a pressure ulcer . . .* [before that] *I knew nothing about them"* (Patti, carer). Information received in advance of pressure ulcer development was generally not remembered. Another hinderance to PU knowledge was that they were considered a "no-go" area, with comments such as "i*t's one of those things I suppose that when you're sitting in the pub having a quiet pint with someone you don't discuss, do you?"* (Mick, patient).

Theme 2: Social and professional role and influences

This theme included barriers relating to participants being confused as to the role of visiting practitioners, advice which appeared to conflict and disagreements between practitioners or practitioners and carers.

Who does what?

Most participants were unclear about what practitioners did, the purpose of their visit and who to approach about PUs. Table 2 illustrates the nature and number of health and social carers with whom individuals were in contact over the previous year. In extreme cases the volume and number of visits left participants reflecting a sense of chaos, for example, Cass (carer), *"four people coming a day, nurses, carers, doctors . . . physios somebody knocking on your door, it's hard . . I've had the physio', occupation therapist . . . they bring the tissue viability nurse and some other ladies . . . two ladies, um, I think they must be physios I think"*. Similarly, Patti (carer) said *"there's an older lady who's very good and she just gets on . . . does whatever is needed. And then this other person will suddenly turn up and you think, why are you here?"*.

For peer review only

Table 2: Community health and social services supporting patients (✓) and carers (*) in the last year

Pseudonym	Role	Carer centre	Day centre	District nurse	Family carer live-in	Family carer visiting	GP	Occupational therapist	Paid carer live-in	Paid carer visiting	Pharmacy	Physiotherapist	Podiatrist	Residential respite	Sitter	Social prescriber	Social worker	Specialist nurse	Speech and language	Tissue viability nurse
George	Patient			✓		✓		✓	✓	✓										
Joni	Patient			✓		✓		✓			✓	✓								
Paul	Patient			✓		✓	✓	✓	✓	✓										
Mick	Patient			✓								✓								
Carly	Patient			✓	✓															
John	Patient			✓	✓		✓	✓												
Tina	Patient			✓	✓															
Bob	Patient			✓		✓	✓	✓		✓	✓	✓	✓			✓				
Marvin	Patient		✓	✓	✓	✓				✓										✓
Linda	Patient			✓	✓	✓	✓				✓	✓		✓						
Cass	Carer			✓	✓	✓	✓	✓		✓		✓	✓		*					
Janis	Carer	*		✓	✓	✓	✓					✓	✓							✓
Nina	Carer	*			✓			✓		✓			✓							
Jimmy	Carer			✓	✓							✓								
Patsy	Carer			✓	✓		✓	✓												
Frank	Carer				✓	✓	✓				✓	✓		*						
Martha	Carer			✓	✓	✓		✓		✓				*			✓			
Glen	Carer	*		✓	✓		✓	✓							✓		✓			
Diana	Carer			✓		✓	✓	✓		✓					✓			✓	✓	
Patti	Carer			✓		✓		✓		✓		✓		*						

For peer review only

Conflicting advice and disagreements

Participants described variations in practitioner advice and being asked to pass instructions from one to another, causing distress and awkwardness. Nina (carer) explained *"we're in the middle, we've got a podiatrist telling us . . . 'will you tell your nurses about it?' Then we've had to relay that to three or four different nurses who see* [husband]*".* Sometimes people reported conflicts between what the professional suggested and what they, their family members or lay carers thought best. Paul (patient) described how his live-in carer reacted when he did not agree with the physiotherapist's advice, *"nearly took her head off . . . he gets a bit agitated, my carer".* On occasion, lay carers' advice took priority over practitioners', *"I take all the advice that* [wife] *ever gives . . . but in general I have faith in the NHS*" (John, patient). Jimmy (carer) described an ongoing battle with district nurses regarding pressure relieving equipment, *"she had oedema . . . incredibly wet and sweaty in the plastic, which I didn't think was a very good idea . . . I kept taking them off and the nurses kept telling me off and putting them back on again".* Sometimes participants were concerned disagreements would negatively impact care, *"you've got to be careful because you don't want to upset these people who are caring for* [husband]" (Nina, carer). Similarly, patients and lay carers did not always agree on care, in these instances the burden of the disagreement seemed more emotionally arduous for both parties. Some carers described how the person cared for found accepting care and adhering to PU prevention behaviours a challenge, "*won't take on board the seriousness of it on and read the information. . .* [he] *would say you're keeping on too much . . . nagging"* (Cass, carer).

Theme 3: Motivation and priorities

Most patients and carers considered PU prevention to be important but there were two key barriers, patients having to prioritise other physical health needs, and the physical restrictions of lay carers.

Competing self-care needs

This theme related in the most part to repositioning. Nearly all participants reported that the patient was physically unable to adopt PU advice due to physical restrictions. Most often cited were breathlessness, pain or fear of falling. *"I tend not to lie flat . . . I'm sitting up higher . . . I do turn on my side, but not fully"* (John, patient). George (patient) said, *"sometimes, I'm in so much pain in my legs I can't do anything".* Inspecting vulnerable areas for deterioration was challenging due to physical restrictions, Cass, carer, speaking about her own vulnerability to PUs found a way around this, she said "*can't see behind . . . I asked my daughter to take a picture".* To a lesser extent, poor health resulted in a reduced appetite, which inhibited good nutrition as a prevention behaviour.

Carer physical ability

For peer review only - http://bmjopen.bmj.com/site/about/guidelines.xhtml

Some carers were restricted by their own health issues, *I have to hoist him to reposition him every time . . . I am very cautious how I move him now because, you know, I was 51 when I started and I'm now 73, there is a lot of difference in that age group"* (Janis, carer).

Theme 4: Memory

Five people cited memory as a barrier, Patsy (carer) said, *"he needs to be reminded* [to eat and drink]*"* and Martha (carer), *"needed to be encouraged and reminded* [to change position], *she would forget".* Two participants suggested alarms as reminders, for example *"setting it up on my phone, a reminder"* (Jimmy, patient).

Theme 5: Emotion

The emotional burden, including exhaustion and isolation, of giving or receiving care was significant irrespective of the presence of PUs or the need for PU prevention. Some participants spoke of conflicting carer versus partner roles and a range of other feelings.

Carer exhaustion and isolation

Carer exhaustion and isolation was indirectly linked with PU prevention and was a problem expressed by all carers and most patients. Carers felt frustrated, alone and in need of practical and emotional support. "*Carers . . . they're left in a hole on their own and all the time they're trying to get out of this hole and deal with everything . . . it takes a lot of emotional and, and, energy"* (Cass, carer). Patsy (carer) was reluctant to speak to or seek support from her grown up children, *"I would hesitate to load too much onto them . . . because they care so much about* [husband]*".* Sometimes carers expressed the caring relationship as "two-way", where support was exchanged, but this was not always the case. Breaks out of the house helped carers to see friends or attend support groups, sometimes making use of "sitters". *"I had a sitting service . . . twice a week . . . a lifesaver. . . just for two hours . . . it doesn't have to be that you go out"* (Cass, carer). Social contact for some was nearly impossible, as Diana (carer) told us, *"if we went somewhere socially, there's not always a disabled toilet and there is no grabrail . . . I miss people".* Emotions impacted on interactions, leading to feelings of guilt, "*there have been* [times] *I have lost the plot . . . I feel awful . . . I have a flare up . . . he says he understands, and he does"* (Nina, carer). Lay carers sometimes found professional care visits an intrusion of privacy and closeness, *"my house was no longer my home, I lost that. And I think we lost a little bit of our closeness"* (Janis, carer). Beyond exhaustion and isolation, carers listed many emotions, for example, fear of their partner dying before them, or of their partner being left behind should they die, guilt that their care was insufficient or inadequate, worries about finances, frustrations of trying to get additional health or social care help, the vulnerability of needing to rely on others and distress at seeing a relative in pain or unwell.

For peer review only - http://bmjopen.bmj.com/site/about/guidelines.xhtml

Carer role versus partner role

Partner carers described personal role conflict and expressed differing opinions about the extent of their carer role. For example, *"I personally didn't help* [PU care], *no, it came through the nurses"* (Cass, carer), in contrast another carer explained *"I don't particularly want anyone to look after him . . . intrusion of our closeness"* (Janis). Being both partner and carer was a concern. Often this was about dignity and not allowing the "partnership" to be subsumed by a caring role. Carers dealt with this by compartmentalising. For example, Cass (carer), *"we have a plan, my husband and I, it's like I'm always going to be his wife, and when I do certain things . . .I say to him, I've got my carer's hat on . . . things that aren't quite so nice . . . I feel I'm two different people".*

Patient feelings

Patients reported malaise, low energy and low mood adding to the challenge of PU prevention. *"The body doesn't last forever . . . it is frustrating. . . can't even do the simplest things . . .life isn't a bed of roses when you get older"* (George, patient). Carers recognised and empathised, for example, "[it's] *sad, this fiercely independent, highly intelligent man is just not able to do things anymore"* (Patsy, carer). Patients were embarrassed by younger formal carers inspecting parts of their body, feelings carers empathised with, *"I mean we laugh and joke about it . . that was my way of dealing with . . . it's embarrassment"* (Mick, patient) and *"I always did what I call his private bits . . . washed and creamed him . . . some of the girls are 17 and 18 years old . . . it's his dignity"* (Diana, carer).

Theme 6: Environment

The key environmental barrier was human resources. Equipment was generally available but at times provided too late.

Human resource and response

Over half of participants spoke about the frustrations of getting help from HCPs or making appointments. "[I] *want to scream . . . you spend half your life on the telephone trying to chase things . . . it takes a lot of emotional . . . and energy . . . nurses not turning up . . . transport doesn't turn up . . . that happens such a lot"* (Cass, carer). Out of hours provision was a problem, *"I dread every bank holiday . . . if something is going to go wrong"* (Janis, carer). Participants believed that practitioners were short of time, short of funds, overworked or stressed, *"overburdened . . . I know they are just overworked, spread too thin"* (Martha, carer).

Equipment

Required equipment was provided, including profiling beds, bed extensions, walking frames, hoists, cushions, offloading wedges, mattresses and handrails. The only concerns were lack of support to

For peer review only - http://bmjopen.bmj.com/site/about/guidelines.xhtml

For peer review only

install it and delays in receiving it. For example, Joni (patient) remembered, *"this man, he said mattress, and he plonked the tubes down. So, I said, well what do I do with them? He said, haven't you got anyone here? I said no. Oh, I'll do it, he said . . . the bloody fool . . . he'd just left it on top* [of bedding]*"*.

Discussion

In interviews underpinned by the TDF[15] with ten patients and ten carers we identified six themes representing barriers to PU prevention behaviours: i) knowledge and beliefs about consequences, ii) social or professional role and influences, iii) motivation and priorities, iv) memory, v) emotion and vi) environment.

Although most barriers identified were individual in nature, one significant barrier, the late acquisition of PU knowledge (*after* PU development), partly attributable to its taboo nature, suggests population level strategies are needed. In cases of similarly taboo areas of health, such as bowel cancer, campaigns have achieved some success. A recent campaign, involving diary style podcasts, led by a young woman with terminal bowel cancer, destigmatised "toilet habits" and led to a tenfold increase in online searches for bowel cancer symptoms immediately following her death[19]. For prostate cancer, there was a tenfold increase in use of a publicly available "30-second checker" prostate screening tool after a television presenter announced his diagnosis[20]. Social media and online campaigns are not accessed by or useful to all[21]. For the populations in question, predominantly older people, this may offer only a partial solution. For example, systematic review evidence reports that 67% of older people access the internet compared with 90% of all adults[22]. In addition, recent research shows that internet campaigns are less effective for men than women[22]. However, such campaigns may have a value in that those accessing may influence others (e.g., women and younger people influencing male and older relatives) and indeed the younger people of today are our future "at risk" generation.

When seeking to address individual barriers there is a need for solutions to be tailored to the individual[23]. The TDF can be used to identify the specific behaviour change techniques (BCTs) that are most likely to be effective[16]. This approach has been used and systematically reviewed in the case of both HCPs[24] and patients and carers[25]. These BCTs can be translated into pragmatic intervention components. A co-design approach involving end users[26] is most likely to result in a product that is acceptable, practicable and equitable[27]. There is published precedent[27], and systematic review guidance[28], for high quality design with older people.

There were strengths and limitations to our study. We used a structured theoretical approach to data collection and analysis. Service users were involved in design, analysis and reporting. Most

For peer review only - http://bmjopen.bmj.com/site/about/guidelines.xhtml

research relating to PUs considers hospital settings and health care practice. To the best of our knowledge this is the first to consider community dwelling older patients and their lay carers. We recruited from only one geographical area, although our results may be transferrable it is possible that elements of service provision may differ both nationally and internationally. Whilst we sought to establish facilitators (as well as barriers) these related only to the provision of equipment and were therefore not helpful in informing future solutions.

HCPs providing care to community dwelling older people need to give timely and consistent PU prevention messages, in an accessible format to patients and lay carers. Greater clarity in roles and responsibility among care providers and across HCP and lay carer boundaries would be valuable.

As our research is, to the best of our knowledge, the first to investigate barriers and facilitators to PU prevention in community dwelling older people, further research is needed. Now we understand the barriers there is an opportunity to co-design strategies to support PU prevention behaviours in older people and test these in practice.

Conclusion

Little research has considered the contribution made by community-dwelling patients and their lay carers in preventing pressure ulcers. This study sought to understand the barriers and facilitators to engaging in pressure ulcer prevention through robust application of the theoretical domains framework. Our findings demonstrate that the key barriers relate to knowledge and beliefs about consequences, social and professional roles and influences, motivation and priorities, memory, emotion, and environment. This knowledge will underpin the co-design of strategies to promote preventative behaviours around pressure ulcer prevention for this population. The learning from this project is likely to be transferable to comparable national and international health care systems.

For peer review only

Funding statement

This project is funded by the National Institute for Health and Care Research (NIHR) under its Research for Patient Benefit (RfPB) Programme (Grant Reference Number NIHR201933). The views expressed are those of the author(s) and not necessarily those of the NIHR or the Department of Health and Social Care.

Competing interests

None declared

Authors contributions

JR, MW, FC, JD, AD and KO designed the study. JR and MW contributed to data collection. JD, MW and JR contributed to data analysis. FC, JD, JR and MW drafted the manuscript. MW, JR, KO and AD critically revised the manuscript. JR is responsible for the overall content as the guarantor, had full access to the data and takes responsibility for the integrity of the data and the accuracy of the data analysis.

Acknowledgements

Dr Carol Samuel collected some of the data for this project and began the data analysis.

Data sharing statement

The dataset for this study is not available at this time.

Word count

3992 (not including figures and tables)

Ethics

This study was reviewed and approved by Cornwall and Plymouth Research Ethics Committee (reference 21/SW/0061).

For peer review only

For peer review only - http://bmjopen.bmj.com/site/about/guidelines.xhtml

References

1. European Pressure Ulcer Advisory Panel, National Pressure Ulcer Advisory Panel, Pan Pacific Pressure Injury Alliance (2019) Prevention and treatment of pressure ulcers/ injuries: Clinical Practice Guideline. The International Guideline.
2. Li Z, Lin F, Thalib L, et al. Global prevalence and incidence of pressure injuries in hospitalised adult patients: A systematic review and meta-analysis. *International journal of nursing studies* 2020;105:103546.
3. Sugathapala RUP, Latimer S, Balasuriya A, et al. Prevalence and incidence of pressure injuries among older people living in nursing homes: A systematic review and Meta-analysis. *International Journal of Nursing Studies* 2023:104605.
4. NHS Digital. Hospital Admitted Patient Care Activity 2017-2018 [Available from: https://digital.nhs.uk/data-and-information/publications/statistical/hospital-admitted-patient-care-activity/2018-192023.
5. Gorecki C, Brown JM, Nelson EA, et al. Impact of pressure ulcers on quality of life in older patients: a systematic review. *Journal of the American Geriatrics Society* 2009;57(7):1175-83.
6. Jackson DE, Durrant LA, Hutchinson M, et al. Living with multiple losses: Insights from patients living with pressure injury. *Collegian* 2018;25(4):409-14.
7. Landi F, Onder G, Russo A, et al. Pressure ulcer and mortality in frail elderly people living in community. *Archives of gerontology and Geriatrics* 2007;44:217-23.
8. Roddis JK, Liversedge HL, Ryder I, et al. Incorporating the patient experience into clinical guidelines: recommendations for researchers and guideline developers. *BMJ Evidence-Based Medicine* 2019;24(4):125-26.
9. James Lind Alliance Priority Setting Partnership. Pressure Ulcers Top 10 [Available from: https://www.jla.nihr.ac.uk/priority-setting-partnerships/pressure-ulcers/top-10-priorities/2023.
10. National Institute for Health and Care Excellence. Pressure ulcers: prevention and management Clinical guideline 2014 [Available from: www.nice.org.uk/guidance/cg1792023.
11. O'Connor T, Moore ZE, Patton D. Patient and lay carer education for preventing pressure ulceration in at-risk populations. *Cochrane Database of Systematic Reviews* 2021(2)
12. MCCOULOUGH S. Implementing an adapted SSKIN bundle and visual aid in the community setting. *Wounds UK* 2016;12(1)
13. Skivington K, Matthews L, Simpson SA, et al. A new framework for developing and evaluating complex interventions: update of Medical Research Council guidance. *bmj* 2021;374
14. National Institute for Health and Care Excellence, Behaviour change: individual approaches Public health guideline [PH49] 2014 [Available from: https://www.nice.org.uk/Guidance/PH49.
15. Michie S, Johnston M, Abraham C, et al. Making psychological theory useful for implementing evidence based practice: a consensus approach. *BMJ Quality & Safety* 2005;14(1):26-33.
16. Michie S, Johnston M, Francis J, et al. From theory to intervention: mapping theoretically derived behavioural determinants to behaviour change techniques. *Applied psychology* 2008;57(4):660-80.
17. Francis JJ, Johnston M, Robertson C, et al. What is an adequate sample size? Operationalising data saturation for theory-based interview studies. *Psychology and health* 2010;25(10):1229-45.
18. Braun V, Clarke V. Using thematic analysis in psychology. *Qualitative research in psychology* 2006;3(2):77-101.
19. NHS England. Lasting gifts of "bowel babe" Dame Deborah continue in East of England 2023 [Available from: https://www.england.nhs.uk/east-of-england/2023/06/28/2023.
20. BBC News. Nick Owen: Prostate cancer charity sees increase in demand 2023 [Available from: https://www.bbc.co.uk/news/uk-england-birmingham-664391222023.

For peer review only - http://bmjopen.bmj.com/site/about/guidelines.xhtml

21. Patel MS, Halpern JA, Desai AS, et al. Success of prostate and testicular cancer awareness campaigns compared to breast cancer awareness month according to internet search volumes: a google trends analysis. *Urology* 2020;139:64-70.
22. Hunsaker A, Hargittai E. A review of Internet use among older adults. *New media & society* 2018;20(10):3937-54.
23. Baker R, Camosso-Stefinovic J, Gillies C, et al. Tailored interventions to address determinants of practice. *Cochrane Database of Systematic Reviews* 2015(4)
24. Dyson J, Cowdell F. How is the Theoretical Domains Framework applied in designing interventions to support healthcare practitioner behaviour change? A systematic review. *International Journal for Quality in Health Care* 2021
25. Cowdell F, Dyson J. How is the Theoretical Domains Framework applied in health behaviour change? A systematic search and narrative synthesis. *PROSPERO International prospective register of systematic reviews* 2018
26. Jackson CL, Greenhalgh T. Co-creation: a new approach to optimising research impact. *Med J Aust* 2015;203(7):283-4.
27. Glover L, Dyson J, Cowdell F, et al. Healthy ageing in a deprived northern UK city: A co-creation study. *Health & Social Care in the Community* 2020;28(6):2233-42.
28. Cowdell F, Dyson J, Sykes M, et al. How and how well have older people been engaged in healthcare intervention design, development or delivery using co-methodologies: A scoping review with narrative summary. *Health & Social Care in the Community* 2020

For peer review only

For peer review only - http://bmjopen.bmj.com/site/about/guidelines.xhtml

Figure 1: Themes and subthemes

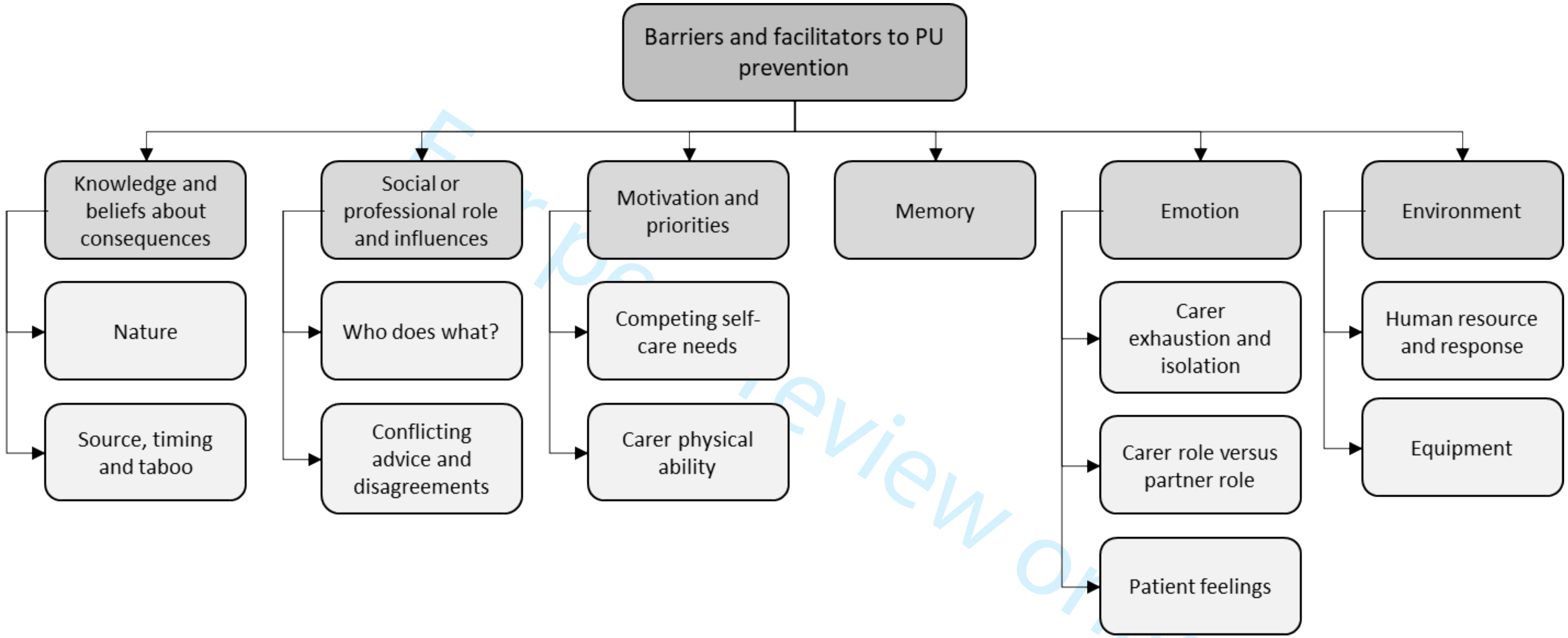

For peer review only - http://bmjopen.bmj.com/site/about/guidelines.xhtml

COREQ (COnsolidated criteria for REporting Qualitative research) Checklist

A checklist of items that should be included in reports of qualitative research. You must report the page number in your manuscript where you consider each of the items listed in this checklist. If you have not included this information, either revise your manuscript accordingly before submitting or note N/A.

Topic	Item No.	Guide Questions/Description	Reported on Page No.
**Domain 1: Research team and reflexivity**			
Personal characteristics			
Interviewer/facilitator	1	Which author/s conducted the interview or focus group?	4
Credentials	2	What were the researcher's credentials? E.g. PhD, MD	4
Occupation	3	What was their occupation at the time of the study?	4
Gender	4	Was the researcher male or female?	4
Experience and training	5	What experience or training did the researcher have?	4
Relationship with participants			
Relationship established	6	Was a relationship established prior to study commencement?	4
Participant knowledge of the interviewer	7	What did the participants know about the researcher? e.g. personal goals, reasons for doing the research	4
Interviewer characteristics	8	What characteristics were reported about the inter viewer/facilitator? e.g. Bias, assumptions, reasons and interests in the research topic	4
**Domain 2: Study design**			
Theoretical framework			
Methodological orientation and Theory	9	What methodological orientation was stated to underpin the study? e.g. grounded theory, discourse analysis, ethnography, phenomenology, content analysis	3
Participant selection			
Sampling	10	How were participants selected? e.g. purposive, convenience, consecutive, snowball	3
Method of approach	11	How were participants approached? e.g. face-to-face, telephone, mail, email	3
Sample size	12	How many participants were in the study?	4
Non-participation	13	How many people refused to participate or dropped out? Reasons?	3
Setting			
Setting of data collection	14	Where was the data collected? e.g. home, clinic, workplace	4
Presence of non-participants	15	Was anyone else present besides the participants and researchers?	4
Description of sample	16	What are the important characteristics of the sample? e.g. demographic data, date	5-6
Data collection			
Interview guide	17	Were questions, prompts, guides provided by the authors? Was it pilot tested?	3
Repeat interviews	18	Were repeat inter views carried out? If yes, how many?	4
Audio/visual recording	19	Did the research use audio or visual recording to collect the data?	4
Field notes	20	Were field notes made during and/or after the inter view or focus group?	4
Duration	21	What was the duration of the inter views or focus group?	7
Data saturation	22	Was data saturation discussed?	4
Transcripts returned	23	Were transcripts returned to participants for comment and/or	4

For peer review only

Topic	Item No.	Guide Questions/Description	Reported on Page No.
		correction?	
**Domain 3: analysis and findings**			
Data analysis			
Number of data coders	24	How many data coders coded the data?	4
Description of the coding tree	25	Did authors provide a description of the coding tree?	4
Derivation of themes	26	Were themes identified in advance or derived from the data?	4
Software	27	What software, if applicable, was used to manage the data?	4
Participant checking	28	Did participants provide feedback on the findings?	4
Reporting			
Quotations presented	29	Were participant quotations presented to illustrate the themes/findings? Was each quotation identified? e.g. participant number	7-13
Data and findings consistent	30	Was there consistency between the data presented and the findings?	7-13
Clarity of major themes	31	Were major themes clearly presented in the findings?	7-13
Clarity of minor themes	32	Is there a description of diverse cases or discussion of minor themes?	11 (theme 4)

Developed from: Tong A, Sainsbury P, Craig J. Consolidated criteria for reporting qualitative research (COREQ): a 32-item checklist for interviews and focus groups. *International Journal for Quality in Health Care*. 2007. Volume 19, Number 6: pp. 349 – 357

Once you have completed this checklist, please save a copy and upload it as part of your submission. DO NOT include this checklist as part of the main manuscript document. It must be uploaded as a separate file.

For peer review only

BMJ Open

Barriers and facilitators to pressure ulcer prevention behaviours by older people and their lay carers in community settings: A qualitative study

Journal:	*BMJ Open*
Manuscript ID	bmjopen-2023-080398.R1
Article Type:	Original research
Date Submitted by the Author:	17-Feb-2024
Complete List of Authors:	Roddis, Jennifer; University of Portsmouth, School of Health and Care Professions<br>Dyson, Judith; Birmingham City University, C-SCHaRR<br>Woodhouse, Marjolein; University of Portsmouth, School of Health and Care Professions<br>Devrell, Anne; Patient and Public Involvement representative<br>Oakley, Karen; Solent NHS Trust<br>Cowdell, Fiona; Birmingham City University, Faculty of Health Education and Life Sciences
**Primary Subject Heading**:	Nursing
Secondary Subject Heading:	Qualitative research
Keywords:	Aging, Nursing Care, Patients, Primary Prevention, QUALITATIVE RESEARCH

SCHOLARONE™
Manuscripts

I, the Submitting Author has the right to grant and does grant on behalf of all authors of the Work (as defined in the below author licence), an exclusive licence and/or a non-exclusive licence for contributions from authors who are: i) UK Crown employees; ii) where BMJ has agreed a CC-BY licence shall apply, and/or iii) in accordance with the terms applicable for US Federal Government officers or employees acting as part of their official duties; on a worldwide, perpetual, irrevocable, royalty-free basis to BMJ Publishing Group Ltd ("BMJ") its licensees and where the relevant Journal is co-owned by BMJ to the co-owners of the Journal, to publish the Work in this journal and any other BMJ products and to exploit all rights, as set out in our licence.

The Submitting Author accepts and understands that any supply made under these terms is made by BMJ to the Submitting Author unless you are acting as an employee on behalf of your employer or a postgraduate student of an affiliated institution which is paying any applicable article publishing charge ("APC") for Open Access articles. Where the Submitting Author wishes to make the Work available on an Open Access basis (and intends to pay the relevant APC), the terms of reuse of such Open Access shall be governed by a Creative Commons licence – details of these licences and which Creative Commons licence will apply to this Work are set out in our licence referred to above.

Other than as permitted in any relevant BMJ Author's Self Archiving Policies, I confirm this Work has not been accepted for publication elsewhere, is not being considered for publication elsewhere and does not duplicate material already published. I confirm all authors consent to publication of this Work and authorise the granting of this licence.

For peer review only

Barriers and facilitators to pressure ulcer prevention behaviours by older people and their lay carers in community settings: A qualitative study

Objective: To identify barriers and facilitators to pressure ulcer (PU) prevention behaviours in community dwelling older people and their lay carers.

Design: Theoretically informed qualitative interviews with two phase, deductive then inductive thematic analysis.

Setting: The study was conducted in one geographical region in the United Kingdom, spanning several community NHS Trusts.

Participants: Community dwelling older patients at risk of pressure ulcer development (n=10) and their lay carers (n=10).

Results: Six themes and subthemes were identified: i) knowledge and beliefs about consequences (nature, source, timing and taboo), ii) social and professional role and influences (who does what, conflicting advice and disagreements), iii) motivation and priorities (competing self-care needs and carer physical ability), iv) memory, v) emotion (carer exhaustion and isolation, carer role versus partner role and patient feelings) and vi) environment (human resource and response, and equipment).

Conclusions: There is minimal research in pressure ulcer prevention in community dwelling older people. This study has robustly applied the theoretical domains framework to understanding barriers and facilitators to pressure ulcer prevention behaviours. Our findings will support co-design of strategies to promote preventative behaviours and are likely to be transferable to comparable health care systems nationally and internationally.

Strengths and limitations

- A structured theoretical approach was adopted to data collection and analysis Both inductive and deductive understandings of barriers and facilitators to PU prevention behaviours were gained, leading to a detailed analysis of influencing factors
- Service users were involved in the design, analysis and reporting of this research
- Recruitment was undertaken in a single geographical area.

For peer review only - http://bmjopen.bmj.com/site/about/guidelines.xhtml

Barriers and facilitators to pressure ulcer prevention behaviours by older people and their lay carers in community settings: A qualitative study

Introduction

Pressure ulcers (PUs) are localised injuries to the skin and underlying tissues, usually occurring over a bony prominence, resulting from pressure, or pressure combined with shear (1). Globally, a PU prevalence of 12.8% and 11.6% has been reported among hospitalised patients (2) and nursing home residents (3), respectively. Pressure ulcers lead to an increased risk of hospital admission, and treatment is costly, placing a substantial burden on care providers (4). PUs have a detrimental impact on the quality of life of affected individuals (5,6) and are associated with increased morbidity, mortality, pain, fear and despondency (6,7). Whilst international guidelines recommend PU prevention behaviours, these focus on a hospital population and there is a dearth of research surrounding transferability to community settings (8). A Priority Setting Partnership involving 500 patients, lay carers and healthcare practitioners (HCP) identified *Understanding the impact of patient and lay carer involvement* as the second most important priority for PU prevention (9).

Existing evidence-based guidelines offer staff intensive PU prevention strategies including i) repositioning at least every four to six hours, ii) use of appropriate devices (e.g., pressure-relieving mattresses), iii) frequent skin inspection and skin care, and iv) optimal nutrition and hydration (10,11). Although these strategies are appropriate in all settings, home care has unique challenges that limit their applicability. Nursing staff and paid carers are able to spend only limited time with patients, so engagement of patients and lay carers in PU prevention is essential. However, there is limited empirical evidence regarding adherence of patients and lay carers to PU prevention guidance. To date, interventions to enhance patient and lay carer PU prevention behaviours have focused on education, though these have had uncertain impact on knowledge or the number of new pressure ulcers (11). This suggests knowledge might not be the only factor for non-adherence (12).

When developing strategies or interventions to support health behaviour change, explicit use of theory allows an understanding of behavioural determinants (barriers and facilitators), thus enabling mapping of empirically tested behaviour change techniques (13,14). The Theoretical Domains Framework (TDF) (15) synthesises all published theories of behaviour or behaviour change into 11 accessible theoretical domains. These domains offer a comprehensive framework of all potential determinants of behaviour (*knowledge, skills, social/professional role and identity, beliefs about capabilities, beliefs about consequences, motivation and goals, memory attention and decision processes, environmental context and resources, social influences, emotion and action planning*).

For peer review only - http://bmjopen.bmj.com/site/about/guidelines.xhtml

Identifying key determinants using the TDF supports identification of the behaviour change techniques (BCTs) most likely to be effective (16).

Objective

To identify barriers and facilitators to PU prevention behaviours in community dwelling older people and their lay carers.

Methods

Design

Qualitative interviews were conducted to elicit barriers and facilitators to PU prevention behaviours. The interview schedule was informed by domains of the TDF.

Participants

Participants were older adults and lay carers. Older adults were community dwelling in their own home, aged ≥65 years, receiving community healthcare and assessed as being at risk of developing a PU (irrespective of current and previous PU status). Lay carers were those providing unpaid care (for people fitting our criteria for older adult participation), of any kind (e.g., physical, household or other practical support). Exclusion criteria included lacking capacity (patients and carers), patients who had not been assessed for pressure ulcer risk, and carers supporting someone who did not fulfil the patient inclusion criteria. We aimed to recruit a minimum of ten patients and ten carers (n=20) which we expected to be sufficient to achieve data saturation given the theoretical underpinning of the study (17).

Patient and public involvement

In this study we had the input of two patient and public involvement colleagues. One contributed to the study design and the other to design, interpretation of data, reading and commenting on results and writing up. We worked closely with the local Carers Centre who advised on and supported recruitment and hosted a dissemination event for the local community.

Recruitment

Patient participants were recruited through HCPs at a community NHS Trust who provided written information about the study. Several patients declined to take part as they felt that the study was not relevant to their circumstances. Carers were recruited through leaflets distributed through a range of outreach events and community settings, for example, carers centre, churches, community centres, voluntary organisations and supermarkets. Snowballing techniques were used to enhance recruitment. Recruitment of carers began in November 2021 and of patients in March 2022, and

For peer review only - http://bmjopen.bmj.com/site/about/guidelines.xhtml

continued until May 2023. Potential participants were provided with information about the project, including the reasons for the study. Interested individuals were contacted by the research team approximately a week later, to discuss the study and decide if they wished to participate.

Data collection

Consent was gained at the start of the interview. Interviews took place in a venue of the participants choice (usually their own home). Interviews were undertaken by female, post-doctoral researchers (CS, MW or JR) who were not known to participants prior to being recruited into the research, and who introduced themselves and their backgrounds at the start of the interviews. Participants were offered a gift voucher or BACS payment post-interview. Single interviews were audio recorded and transcribed verbatim. Throughout most interviews, only the researcher and participant were present, though on occasion the person cared for, or the carer were present. One interview was undertaken jointly with the patient and their carer. Notes were taken by the researcher as prompts, these did not form part of the analysis. Transcripts were not returned to participants for comment due to the potentially onerous nature of such checking, particularly with this group for whom it could be considered unethical given the time commitments of carers in particular.

Data analysis

Transcripts were imported into NVivo (v12) and underwent a two-stage analysis. After deductively categorising data to the domains of the TDF, inductive thematic analysis was undertaken according to a six-step process (familiarisation, code generation, combining codes to themes, reviewing themes, determining theme significance and reporting) (18). Data analysis and collection were iterative processes and continued until saturation was achieved. Analysis was initially undertaken by JD, MW and JR and reviewed by the author team.

Results

Characteristics of participants

Table 1 presents participant characteristics (n=20). Patient participants (women n=4, men n=6) were aged 68 to 94 years (mean age 79.5). Three had a current PU, four had previously had PU(s), two had never had a PU and one currently had and had previously experienced PUs. Where patient participants had experience of a PU, five had developed these in hospital and three at home. All patient participants had limited mobility. Lay carer participants (women n=7, men n=3) supported or had supported older relatives, 80% of these participants cared for someone who had a PU. Ages ranged from 56 to 82 years (mean 75.0). Eight lay carers were caring for their partner (husband or wife), one was caring for their mother-in-law and father-in-law, and one for their mother.

For peer review only - http://bmjopen.bmj.com/site/about/guidelines.xhtml

Table 1: Participant Characteristics[1]

Pseudo nym	Role	Gender	Age	Occupation	Health and self-reported mobility status of patient	Self-reported health status of lay carer	Patient PU status
George	Patient	Male	68	Retired teacher	Lower body paralysis, wheelchair to mobilise		Previous hospital acquired sacral PU
Joni	Patient	Female	94	Retired shop worker	Multiple long-term conditions (LTCs), poor mobility, mobilises using a walking frame		Previous hospital acquired sacral PU
Paul	Patient	Male	72	Retired car manufacturer	Paralysis of legs, wheelchair to mobilise		Current hospital acquired sacral PU
Mick	Patient	Male	81	Retired from the armed forces	Usually mobile, currently compromised mobility post-surgery		Current hospital acquired sacral PU
Carly	Patient	Female	67	Retired teacher	Limited mobility using aids (walking frame, stairlift, rails, mobility scooter)		Previous home acquired sacral and foot PUs
John[1]	Patient	Male	92	Retired engineer	Multiple LTCs, usually mobile with a recent period of immobility		Previous home acquired sacral PU
Tina[2]	Patient	Female	74	Retired secretary	Walks short distances and uses a wheelchair		Nil
Bob	Patient	Male	90	Retired joiner	Multiple LTCs, walks at home with frame and two people, otherwise wheelchair user		Nil
Marvin[3]	Patient	Male	72	Retired police officer	Paraplegic, wheelchair user		Current home acquired sacral PU and previous hospital acquired sacral and hip PUs
Linda[4]	Patient	Female	75	Childcare/ nursery	Multiple LTCs, wheelchair user		Current home acquired sacral PU

[1] Where patient and carer were related this is indicated by superscript matching numbers next to participant pseudonym.

For peer review only - http://bmjopen.bmj.com/site/about/guidelines.xhtml

Pseudo nym	Role	Gender	Age	Occupation	Health and self-reported mobility status of patient	Self-reported health status of lay carer	Patient PU status
Cass	Wife caring for husband (now deceased)	Female	81	Retired administrator	Immobile	Osteoporosis	Current home and hospital acquired sacral PUs
Janis[3]	Wife caring for husband	Female	73	Retired administrator	Multiple LTCs and progressive paralysis	Good physical health	Current home acquired sacral PU and previous hospital acquired sacral and hip Pus
Nina	Wife caring for husband	Female	82	Retired administrator	Multiple LTCs, mobile with frame and support, uses wheelchair outdoors	Good physical health	Nil
Jimmy[2]	Husband caring for wife	Male	74	Retired mechanical engineer	Multiple LTCs, limited mobility	Good physical health	Nil
Patsy[1]	Wife caring for husband	Female	81	Retired Nurse	Multiple LTCs, limited mobility	Severe arthritis	Previous hospital acquired sacral PU
Frank[4]	Husband caring for wife	Male	78	Retired Educator	Multiple LTCs, limited mobility	Good physical health	Current home acquired sacral PU
Martha	Daughter-in-law caring for mother and father-in-law (now in care home)	Female	56	Dentist	Both have multiple LTCs and mobilise indoors only with walking frames	Good physical health	1. Home acquired PU lower back/spine 2. nil.
Glen	Son caring for mother	Male	69	Retired surveyor	Limited mobility indoors	Good physical health	Current hospital acquired foot PU
Diana	Wife caring for husband	Female	82	Retired Civil Servant	Multiple LTCs, limited mobility with frame	Arthritis	Previous home acquired PU (site NR)
Patti	Wife caring for husband	Female	74	Retired Teacher	Paralysis, usually wheelchair bound, currently bedbound.	Good physical health	Current hospital acquired multiple site PUs

For peer review only - http://bmjopen.bmj.com/site/about/guidelines.xhtml

For peer review only

Findings

Interviews took a mean of 50 minutes. Six themes (domains of the TDF) and 10 sub-themes were identified (figure 1). Few differences were identified between the barriers and facilitators to PU prevention experienced by carers when compared to those experienced by patients.

Figure 1

Theme 1: Knowledge and beliefs about consequences

Participants generally knew about the strategies to prevent PUs, except for good hydration and nutrition. This knowledge was generated from multiple sources however, in most cases was acquired *after* PU development. The taboo nature of the subject hindered *pre*-PU awareness.

Nature of knowledge

Some found the term pressure ulcer unfamiliar, for example *"it's naivety in that I thought pressure sores were bed sores, so, I thought you only got them in bed"* (Marvin, patient). The three PU prevention strategies known to participants included repositioning and risk of friction when moving, for example, Patsy (carer) stated, *"caused by people spending too much time in one position . . . it limits the blood supply to the skin in that area"*. Many were aware of vulnerable areas particularly referring to the "bottom" with some mentioning back, hips, heels and elbows. Devices were mentioned by most and many used aids to enhance mobility such as frames and 'grab rails' (a bar attached to a wall, usually next to a bath or toilet, which the individual can hold to help them get up or down). Patient and lay carer participants reported inspecting at-risk areas of the skin and raising concerns with HCPs. Although some participants appreciated the value of good hydration and nutrition, this was rarely linked to prevention of PUs.

Source, timing and taboo

The main source of knowledge for participants was HCPs (in hospital or at home), sometimes because they gave advice but often because the participant observed what they did and adopted these behaviours. Pete (patient) told us *"I've never received any information . . . it's really information I overhear from things like nurses . . . I've looked it up on the internet ."* Some participants had received leaflets from HCPs but found them of limited value, for example, "*if you get a wad of paper the feeling immediately is to dismiss it"* (George, patient). Other knowledge sources included Carers' Centre courses and support groups. The words of Cass (carer) captured the

For peer review only - http://bmjopen.bmj.com/site/about/guidelines.xhtml

general consensus *"there definitely needs to be more information out there"* and many highlighted their lack of ability to access online information.

Participants learned about PUs *after* they or their relative had developed one. For example, *"the ward did initially* [told me about PUs], *. . . this is what we call a pressure ulcer . . .* [before that] *I knew nothing about them"* (Patti, carer). Information received in advance of pressure ulcer development was generally not remembered. Another hinderance to PU knowledge was that they were considered unsuitable or too sensitive for discussion (a "no-go" area), with comments such as "*it's one of those things I suppose that when you're sitting in the pub having a quiet pint with someone you don't discuss, do you?"* (Mick, patient).

Theme 2: Social and professional role and influences

This theme included barriers relating to participants being confused as to the role of visiting practitioners, advice which appeared to conflict and disagreements between practitioners or practitioners and carers.

Who does what?

Most participants were unclear about what practitioners did, the purpose of their visit and who to approach about PUs. Supplementary Table 1 illustrates the nature and number of health and social carers with whom individuals were in contact over the previous year. In extreme cases the volume and number of visits left participants reflecting a sense of chaos, for example, Cass (carer), *"four people coming a day, nurses, carers, doctors . . . physios somebody knocking on your door, it's hard . . I've had the physio', occupation therapist . . . they bring the tissue viability nurse and some other ladies . . . two ladies, um, I think they must be physios I think"*. Similarly, Patti (carer) said *"there's an older lady who's very good and she just gets on . . . does whatever is needed. And then this other person will suddenly turn up and you think, why are you here?"*.

Supplementary table 1

For peer review only - http://bmjopen.bmj.com/site/about/guidelines.xhtml

Conflicting advice and disagreements

Participants described variations in practitioner advice and being asked to pass instructions from one to another, causing distress and awkwardness. Nina (carer) explained *"we're in the middle, we've got a podiatrist telling us . . . 'will you tell your nurses about it?' Then we've had to relay that to three or four different nurses who see* [husband]*".* Sometimes people reported conflicts between what the professional suggested and what they, their family members or lay carers thought best. Paul (patient) described how his live-in carer reacted when he did not agree with the physiotherapist's advice, *"nearly took her head off . . . he gets a bit agitated, my carer".* On occasion, lay carers' advice took priority over practitioners', *"I take all the advice that* [wife] *ever gives . . . but in general I have faith in the NHS*" (John, patient). Jimmy (carer) described an ongoing battle with district nurses regarding pressure relieving equipment, *"she had oedema . . . incredibly wet and sweaty in the plastic, which I didn't think was a very good idea . . . I kept taking them off and the nurses kept telling me off and putting them back on again".* Sometimes participants were concerned disagreements would negatively impact care, *"you've got to be careful because you don't want to upset these people who are caring for* [husband]" (Nina, carer). Similarly, patients and lay carers did not always agree on care, in these instances the burden of the disagreement seemed more emotionally arduous for both parties. Some carers described how the person cared for found accepting care and adhering to PU prevention behaviours a challenge, "*won't take on board the seriousness of it on and read the information. . .* [he] *would say you're keeping on too much . . . nagging"* (Cass, carer).

Theme 3: Motivation and priorities

Most patients and carers considered PU prevention to be important but there were two key barriers, patients having to prioritise other physical health needs, and the physical restrictions of lay carers.

Competing self-care needs

This theme related in the most part to repositioning. Nearly all participants reported that the patient was physically unable to adopt PU advice due to physical restrictions. Most often cited were breathlessness, pain or fear of falling. *"I tend not to lie flat . . . I do turn on my side, but not fully"* (John, patient). George (patient) said, *"sometimes, I'm in so much pain in my legs I can't do anything"*. Inspecting vulnerable areas for deterioration was challenging due to physical restrictions, Cass, carer, speaking about her own vulnerability to PUs found a way around this, she said "*can't see behind . . . I asked my daughter to take a picture".* To a lesser extent, poor health resulted in a reduced appetite, which inhibited good nutrition as a prevention behaviour.

Carer physical ability

For peer review only - http://bmjopen.bmj.com/site/about/guidelines.xhtml

Whilst motivated to provide the best possible care for the person they cared for, some carers were restricted by, and thus had to prioritise, their own health issues, *"I have to hoist him to reposition him every time . . . I am very cautious how I move him now because, you know, I was 51 when I started and I'm now 73"* (Janis, carer).

Theme 4: Memory

Five people cited memory as a barrier, Patsy (carer) said, *"he needs to be reminded* [to eat and drink]*"* and Martha (carer), *"needed to be encouraged and reminded* [to change position], *she would forget".* Two participants suggested alarms as reminders, for example *"setting it up on my phone, a reminder"* (Jimmy, patient).

Theme 5: Emotion

The emotional burden, including exhaustion and isolation, of giving or receiving care was significant irrespective of the presence of PUs or the need for PU prevention. Some participants spoke of conflicting carer versus partner roles and a range of other feelings.

Carer exhaustion and isolation

Carer exhaustion and isolation was indirectly linked with PU prevention and was a problem expressed by all carers and most patients. Carers felt frustrated, alone and in need of practical and emotional support. "*Carers . . . they're left in a hole on their own and all the time they're trying to get out of this hole and deal with everything . . . it takes a lot of emotional and, and, energy"* (Cass, carer). Patsy (carer) was reluctant to speak to or seek support from her grown up children, *"I would hesitate to load too much onto them . . . because they care so much about* [husband]*".* Sometimes carers described a mutual "two-way" caring relationship, where support was exchanged, but this was not always the case. Spending time away from the home helped carers to see friends or attend support groups, sometimes making use of "sitters" (a form of respite care where someone from a care company or other organisation comes to the home and spends time with the person being cared for). *"I had a sitting service . . . twice a week . . . a lifesaver. . . it doesn't have to be that you go out"* (Cass, carer). Social contact for some was nearly impossible, as Diana (carer) told us, *"if we went somewhere socially, there's not always a disabled toilet and there is no grabrail . . . I miss people".* Emotions impacted on interactions, leading to feelings of guilt, "*there have been* [times] *I have lost the plot . . . I feel awful . . . I have a flare up . . . he says he understands, and he does"* (Nina, carer). Lay carers sometimes found professional care visits an intrusion of privacy and closeness, *"my house was no longer my home, I lost that. And I think we lost a little bit of our closeness"* (Janis, carer). Beyond exhaustion and isolation, carers listed many emotions, for example, fear of their partner dying before them, or of their partner being left behind should they die, guilt that their care

For peer review only - http://bmjopen.bmj.com/site/about/guidelines.xhtml

was insufficient or inadequate, worries about finances, frustrations of trying to get additional health or social care help, the vulnerability of needing to rely on others and distress at seeing a relative in pain or unwell.

Caregiver role conflict

Partner carers described personal role conflict and expressed differing opinions about the extent of their carer role. For example, *"I personally didn't help* [PU care], *no, it came through the nurses"* (Cass, carer), in contrast another carer explained *"I don't particularly want anyone to look after him . . . intrusion of our closeness"* (Janis). Being both partner and carer was a concern. Often this was about dignity and not allowing the "partnership" to be subsumed by a caring role. Carers dealt with this by compartmentalising. For example, Cass (carer), *"we have a plan, my husband and I, it's like I'm always going to be his wife, and when I do certain things . . .I say to him, I've got my carer's hat on . . . things that aren't quite so nice . . . I feel I'm two different people".*

Patient feelings

Patients reported malaise, low energy and low mood adding to the challenge of PU prevention. *"The body doesn't last forever . . . it is frustrating. . . can't even do the simplest things . . .life isn't a bed of roses when you get older"* (George, patient). Carers recognised and empathised, for example, "[it's] *sad, this fiercely independent, highly intelligent man is just not able to do things anymore"* (Patsy, carer). Patients were embarrassed by younger formal carers inspecting parts of their body, feelings carers empathised with, *"I mean we laugh and joke about it . . that was my way of dealing with . . . it's embarrassment"* (Mick, patient) and *"I always did what I call his private bits . . . washed and creamed him . . . some of the girls are 17 and 18 years old . . . it's his dignity"* (Diana, carer).

Theme 6: Environment

The key environmental barrier was human resources. Equipment was generally available but at times provided too late.

Human resource shortage and heavy burden of care

Over half of participants spoke about the frustrations of getting help from HCPs or making appointments. "[I] *want to scream . . . you spend half your life on the telephone trying to chase things . . . it takes a lot of emotional . . . and energy . . . nurses not turning up . . . transport doesn't turn up . . . that happens such a lot"* (Cass, carer). Out of hours provision was a problem, *"I dread every bank holiday . . . if something is going to go wrong"* (Janis, carer). Participants believed that practitioners were short of time, short of funds, overworked or stressed, *"overburdened . . . I know they are just overworked, spread too thin"* (Martha, carer).

For peer review only - http://bmjopen.bmj.com/site/about/guidelines.xhtml

Equipment

Equipment considered necessary by HCPs caring for patient participants was provided, including profiling beds, bed extensions, walking frames, hoists, cushions, offloading wedges, mattresses and handrails. The only concerns were lack of support to install it and delays in receiving it. For example, Joni (patient) remembered, *"this man, he said mattress, and he plonked the tubes down. So, I said, well what do I do with them? He said, haven't you got anyone here? I said no. Oh, I'll do it, he said . . . the bloody fool . . . he'd just left it on top* [of bedding]*".*

Discussion

In interviews underpinned by the TDF (15) with ten patients and ten carers we identified six themes representing barriers to PU prevention behaviours: i) knowledge and beliefs about consequences, ii) social or professional role and influences, iii) motivation and priorities, iv) memory, v) emotion and vi) environment.

Although most barriers identified were individual in nature, one significant barrier, the late acquisition of PU knowledge (*after* PU development), partly attributable to its taboo nature, suggests population level strategies are needed. In cases of similarly taboo areas of health, such as bowel cancer, campaigns have achieved some success. A recent campaign, involving diary style podcasts, led by a young woman with terminal bowel cancer, destigmatised "toilet habits" and led to a tenfold increase in online searches for bowel cancer symptoms immediately following her death (19). For prostate cancer, there was a tenfold increase in use of a publicly available "30-second checker" prostate screening tool after a television presenter announced his diagnosis (20). Social media and online campaigns are not accessed by or useful to all (21). For the populations in question, predominantly older people, this may offer only a partial solution. For example, systematic review evidence reports that 67% of older people access the internet compared with 90% of all adults (22). In addition, recent research shows that internet campaigns are less effective for men than women (22). We were unable to identify any research evaluating the outcome of the annual European Pressure Ulcer Advisory Panel (EPUAP) 'Stop Pressure Ulcers' campaign designed to raise awareness of PUs. However, such campaigns may have a value in that those accessing may influence others (e.g., women and younger people influencing male and older relatives) and indeed the younger people of today are our future "at risk" generation.

When seeking to address individual barriers there is a need for solutions to be tailored to the individual (23). The TDF can be used to identify the specific behaviour change techniques (BCTs) that are most likely to be effective (16). This approach has been used and systematically reviewed in the case of both HCPs (24) and patients and carers (25). These BCTs can be translated into pragmatic

For peer review only - http://bmjopen.bmj.com/site/about/guidelines.xhtml

intervention components. For example, where knowledge is lacking, information may be the BCT of choice, where motivation is in deficit, BCTs such as goal setting, rewards and consideration of pros and cons to the behaviour in question may offer support (16). A co-design approach involving end users (26) is most likely to result in a product that is acceptable, practicable and equitable (27). There is published precedent (27), and systematic review guidance (28), for high quality design with older people. Elements of co-design are used in the United Kingdom the National Wound Care Strategy Programme in which the Patient Experience Network have created resources by patients for patients. However, to date focus has been on wound care rather than specifically pressure ulcers.

Strengths and limitations

There were strengths and limitations to our study. We used a structured theoretical approach to data collection and analysis. Service users were involved in design, analysis and reporting. Most research relating to PUs considers hospital settings and health care practice. To the best of our knowledge this is the first to consider community dwelling older patients and their lay carers. We recruited from only one geographical area, although our results may be transferrable it is possible that elements of service provision may differ both nationally and internationally. Whilst we sought to establish facilitators (as well as barriers) these related only to the provision of equipment and were therefore not helpful in informing future solutions.

HCPs providing care to community dwelling older people need to give timely and consistent PU prevention messages, in an accessible format to patients and lay carers. Greater clarity in roles and responsibility among care providers and across HCP and lay carer boundaries would be valuable.

As our research is, to the best of our knowledge, the first to investigate barriers and facilitators to PU prevention in community dwelling older people, further research is needed. Now we understand the barriers there is an opportunity to co-design strategies to support PU prevention behaviours in older people and test these in practice.

Conclusion

Little research has considered the contribution made by community-dwelling patients and their lay carers in preventing pressure ulcers. This study sought to understand the barriers and facilitators to engaging in pressure ulcer prevention through robust application of the theoretical domains framework. Our findings demonstrate that the key barriers relate to knowledge and beliefs about consequences, social and professional roles and influences, motivation and priorities, memory, emotion, and environment. This knowledge will underpin further research required to co-design

For peer review only - http://bmjopen.bmj.com/site/about/guidelines.xhtml

For peer review only

strategies to promote preventative behaviours around pressure ulcer prevention for this population. Recommendations for clinical practice include ensuring that patients and their lay carers are provided with consistent, early information about PU prevention strategies and that carers are offered support. The learning from this project is likely to be transferable to comparable national and international health care systems.

Funding statement

This project is funded by the National Institute for Health and Care Research (NIHR) under its Research for Patient Benefit (RfPB) Programme (Grant Reference Number NIHR201933). The views expressed are those of the author(s) and not necessarily those of the NIHR or the Department of Health and Social Care.

Competing interests

None declared

Authors contributions

JR, MW, FC, JD, AD and KO designed the study. JR and MW contributed to data collection. JD, MW and JR contributed to data analysis. FC, JD, JR and MW drafted the manuscript. MW, JR, KO and AD critically revised the manuscript. JR is responsible for the overall content as the guarantor, had full access to the data and takes responsibility for the integrity of the data and the accuracy of the data analysis.

Acknowledgements

Dr Carol Samuel collected some of the data for this project and began the data analysis.

Data sharing statement

Data are available upon reasonable request, from the corresponding author.

Word count

4296 (not including figures and tables)

Ethics

This study was reviewed and approved by Cornwall and Plymouth Research Ethics Committee (reference 21/SW/0061).

For peer review only

For peer review only - http://bmjopen.bmj.com/site/about/guidelines.xhtml

References

1. European Pressure Ulcer Advisory Panel, National Pressure Ulcer Advisory Panel, Pan Pacific Pressure Injury Alliance (2019) Prevention and treatment of pressure ulcers/ injuries: Clinical Practice Guideline. The International Guideline.
2. Li Z, Lin F, Thalib L, et al. Global prevalence and incidence of pressure injuries in hospitalised adult patients: A systematic review and meta-analysis. *International journal of nursing studies* 2020;105:103546.
3. Sugathapala RUP, Latimer S, Balasuriya A, et al. Prevalence and incidence of pressure injuries among older people living in nursing homes: A systematic review and Meta-analysis. *International Journal of Nursing Studies* 2023:104605.
4. NHS Digital. Hospital Admitted Patient Care Activity 2017-2018 [Available from: https://digital.nhs.uk/data-and-information/publications/statistical/hospital-admitted-patient-care-activity/2018-192023.
5. Gorecki C, Brown JM, Nelson EA, et al. Impact of pressure ulcers on quality of life in older patients: a systematic review. *Journal of the American Geriatrics Society* 2009;57(7):1175-83.
6. Jackson DE, Durrant LA, Hutchinson M, et al. Living with multiple losses: Insights from patients living with pressure injury. *Collegian* 2018;25(4):409-14.
7. Landi F, Onder G, Russo A, et al. Pressure ulcer and mortality in frail elderly people living in community. *Archives of gerontology and Geriatrics* 2007;44:217-23.
8. Roddis JK, Liversedge HL, Ryder I, et al. Incorporating the patient experience into clinical guidelines: recommendations for researchers and guideline developers. *BMJ Evidence-Based Medicine* 2019;24(4):125-26.
9. James Lind Alliance Priority Setting Partnership. Pressure Ulcers Top 10 [Available from: https://www.jla.nihr.ac.uk/priority-setting-partnerships/pressure-ulcers/top-10-priorities/2023.
10. National Institute for Health and Care Excellence. Pressure ulcers: prevention and management Clinical guideline 2014 [Available from: www.nice.org.uk/guidance/cg1792023.
11. O'Connor T, Moore ZE, Patton D. Patient and lay carer education for preventing pressure ulceration in at-risk populations. *Cochrane Database of Systematic Reviews* 2021(2)
12. MCCOULOUGH S. Implementing an adapted SSKIN bundle and visual aid in the community setting. *Wounds UK* 2016;12(1)
13. Skivington K, Matthews L, Simpson SA, et al. A new framework for developing and evaluating complex interventions: update of Medical Research Council guidance. *bmj* 2021;374
14. National Institute for Health and Care Excellence, Behaviour change: individual approaches Public health guideline [PH49] 2014 [Available from: https://www.nice.org.uk/Guidance/PH49.
15. Michie S, Johnston M, Abraham C, et al. Making psychological theory useful for implementing evidence based practice: a consensus approach. *BMJ Quality & Safety* 2005;14(1):26-33.
16. Michie S, Johnston M, Francis J, et al. From theory to intervention: mapping theoretically derived behavioural determinants to behaviour change techniques. *Applied psychology* 2008;57(4):660-80.
17. Francis JJ, Johnston M, Robertson C, et al. What is an adequate sample size? Operationalising data saturation for theory-based interview studies. *Psychology and health* 2010;25(10):1229-45.
18. Braun V, Clarke V. Using thematic analysis in psychology. *Qualitative research in psychology* 2006;3(2):77-101.
19. NHS England. Lasting gifts of "bowel babe" Dame Deborah continue in East of England 2023 [Available from: https://www.england.nhs.uk/east-of-england/2023/06/28/2023.
20. BBC News. Nick Owen: Prostate cancer charity sees increase in demand 2023 [Available from: https://www.bbc.co.uk/news/uk-england-birmingham-664391222023.

For peer review only - http://bmjopen.bmj.com/site/about/guidelines.xhtml

21. Patel MS, Halpern JA, Desai AS, et al. Success of prostate and testicular cancer awareness campaigns compared to breast cancer awareness month according to internet search volumes: a google trends analysis. *Urology* 2020;139:64-70.
22. Hunsaker A, Hargittai E. A review of Internet use among older adults. *New media & society* 2018;20(10):3937-54.
23. Baker R, Camosso-Stefinovic J, Gillies C, et al. Tailored interventions to address determinants of practice. *Cochrane Database of Systematic Reviews* 2015(4)
24. Dyson J, Cowdell F. How is the Theoretical Domains Framework applied in designing interventions to support healthcare practitioner behaviour change? A systematic review. *International Journal for Quality in Health Care* 2021
25. Cowdell F, Dyson J. How is the Theoretical Domains Framework applied in health behaviour change? A systematic search and narrative synthesis. *PROSPERO International prospective register of systematic reviews* 2018
26. Jackson CL, Greenhalgh T. Co-creation: a new approach to optimising research impact. *Med J Aust* 2015;203(7):283-4.
27. Glover L, Dyson J, Cowdell F, et al. Healthy ageing in a deprived northern UK city: A co-creation study. *Health & Social Care in the Community* 2020;28(6):2233-42.
28. Cowdell F, Dyson J, Sykes M, et al. How and how well have older people been engaged in healthcare intervention design, development or delivery using co-methodologies: A scoping review with narrative summary. *Health & Social Care in the Community* 2020

For peer review only

For peer review only - http://bmjopen.bmj.com/site/about/guidelines.xhtml

Figure Legend:

Figure 1 – Themes and subthemes

For peer review only

For peer review only - http://bmjopen.bmj.com/site/about/guidelines.xhtml

Figure 1: Themes and subthemes

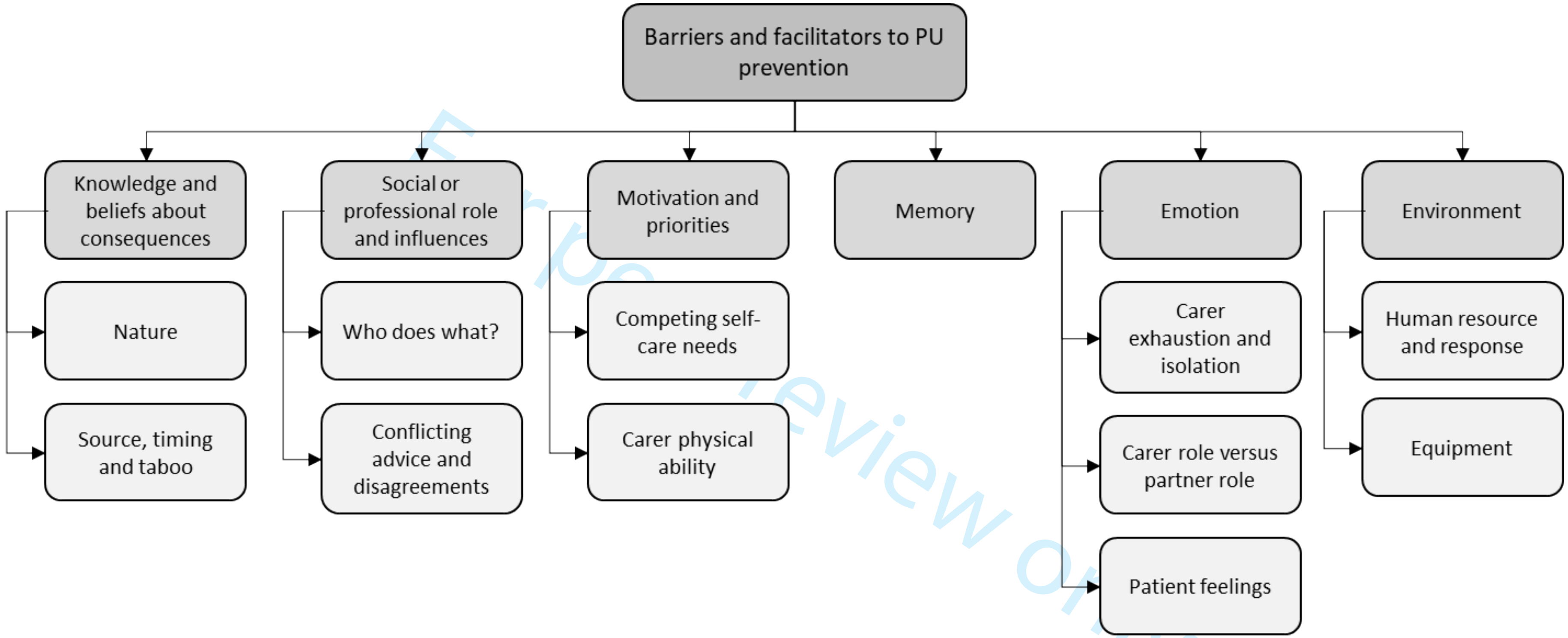

For peer review only - http://bmjopen.bmj.com/site/about/guidelines.xhtml

Supplementary Table 1: Community health and social services supporting patients (✔) and carers (*) in the last year

Pseudonym	Role	Carer centre	Day centre	District nurse	Family carer live-in	Family carer visiting	GP	Occupational therapist	Paid carer live-in	Paid carer visiting	Pharmacy	Physiotherapist	Podiatrist	Residential respite	Sitter	Social prescriber	Social worker	Specialist nurse	Speech and language	Tissue viability nurse
George	Patient			✔		✔		✔	✔	✔										
Joni	Patient			✔		✔		✔			✔	✔								
Paul	Patient			✔		✔	✔	✔	✔	✔										
Mick	Patient			✔								✔								
Carly	Patient			✔	✔															
John	Patient			✔	✔		✔	✔												
Tina	Patient			✔	✔															
Bob	Patient			✔		✔	✔	✔		✔	✔	✔	✔			✔				
Marvin	Patient		✔	✔	✔	✔				✔										✔
Linda	Patient			✔	✔	✔	✔				✔	✔		✔						
Cass	Carer			✔	✔	✔	✔	✔		✔		✔	✔		*					
Janis	Carer	*		✔	✔	✔	✔					✔	✔							✔
Nina	Carer	*			✔			✔		✔			✔							
Jimmy	Carer			✔	✔							✔								
Patsy	Carer			✔	✔		✔	✔												
Frank	Carer				✔	✔	✔				✔	✔		*						
Martha	Carer			✔	✔	✔		✔		✔				*			✔			
Glen	Carer	*		✔	✔		✔	✔							✔		✔			
Diana	Carer			✔		✔	✔	✔		✔					✔			✔	✔	
Patti	Carer			✔		✔		✔		✔		✔		*						

For peer review only

COREQ (COnsolidated criteria for REporting Qualitative research) Checklist

A checklist of items that should be included in reports of qualitative research. You must report the page number in your manuscript where you consider each of the items listed in this checklist. If you have not included this information, either revise your manuscript accordingly before submitting or note N/A.

Topic	Item No.	Guide Questions/Description	Reported on Page No.
**Domain 1: Research team and reflexivity**			
Personal characteristics			
Interviewer/facilitator	1	Which author/s conducted the interview or focus group?	4
Credentials	2	What were the researcher's credentials? E.g. PhD, MD	4
Occupation	3	What was their occupation at the time of the study?	4
Gender	4	Was the researcher male or female?	4
Experience and training	5	What experience or training did the researcher have?	4
Relationship with participants			
Relationship established	6	Was a relationship established prior to study commencement?	4
Participant knowledge of the interviewer	7	What did the participants know about the researcher? e.g. personal goals, reasons for doing the research	4
Interviewer characteristics	8	What characteristics were reported about the inter viewer/facilitator? e.g. Bias, assumptions, reasons and interests in the research topic	4
**Domain 2: Study design**			
Theoretical framework			
Methodological orientation and Theory	9	What methodological orientation was stated to underpin the study? e.g. grounded theory, discourse analysis, ethnography, phenomenology, content analysis	3
Participant selection			
Sampling	10	How were participants selected? e.g. purposive, convenience, consecutive, snowball	3
Method of approach	11	How were participants approached? e.g. face-to-face, telephone, mail, email	3
Sample size	12	How many participants were in the study?	4
Non-participation	13	How many people refused to participate or dropped out? Reasons?	3
Setting			
Setting of data collection	14	Where was the data collected? e.g. home, clinic, workplace	4
Presence of non-participants	15	Was anyone else present besides the participants and researchers?	4
Description of sample	16	What are the important characteristics of the sample? e.g. demographic data, date	5-6
Data collection			
Interview guide	17	Were questions, prompts, guides provided by the authors? Was it pilot tested?	3
Repeat interviews	18	Were repeat inter views carried out? If yes, how many?	4
Audio/visual recording	19	Did the research use audio or visual recording to collect the data?	4
Field notes	20	Were field notes made during and/or after the inter view or focus group?	4
Duration	21	What was the duration of the inter views or focus group?	7
Data saturation	22	Was data saturation discussed?	4
Transcripts returned	23	Were transcripts returned to participants for comment and/or	4

For peer review only

Topic	Item No.	Guide Questions/Description	Reported on Page No.
		correction?	
**Domain 3: analysis and findings**			
Data analysis			
Number of data coders	24	How many data coders coded the data?	4
Description of the coding tree	25	Did authors provide a description of the coding tree?	4
Derivation of themes	26	Were themes identified in advance or derived from the data?	4
Software	27	What software, if applicable, was used to manage the data?	4
Participant checking	28	Did participants provide feedback on the findings?	4
Reporting			
Quotations presented	29	Were participant quotations presented to illustrate the themes/findings? Was each quotation identified? e.g. participant number	7-12
Data and findings consistent	30	Was there consistency between the data presented and the findings?	7-12
Clarity of major themes	31	Were major themes clearly presented in the findings?	7-12
Clarity of minor themes	32	Is there a description of diverse cases or discussion of minor themes?	10 (theme 4)

Developed from: Tong A, Sainsbury P, Craig J. Consolidated criteria for reporting qualitative research (COREQ): a 32-item checklist for interviews and focus groups. *International Journal for Quality in Health Care*. 2007. Volume 19, Number 6: pp. 349 – 357

Once you have completed this checklist, please save a copy and upload it as part of your submission. DO NOT include this checklist as part of the main manuscript document. It must be uploaded as a separate file.

For peer review only

BMJ Open

Barriers and facilitators to pressure ulcer prevention behaviours by older people living in their own homes and their lay carers: A qualitative study

Journal:	*BMJ Open*
Manuscript ID	bmjopen-2023-080398.R2
Article Type:	Original research
Date Submitted by the Author:	01-Mar-2024
Complete List of Authors:	Roddis, Jennifer; University of Portsmouth, School of Health and Care Professions<br>Dyson, Judith; Birmingham City University, C-SCHaRR<br>Woodhouse, Marjolein; University of Portsmouth, School of Health and Care Professions<br>Devrell, Anne; Patient and Public Involvement representative<br>Oakley, Karen; Solent NHS Trust<br>Cowdell, Fiona; Birmingham City University, Faculty of Health Education and Life Sciences
**Primary Subject Heading**:	Nursing
Secondary Subject Heading:	Qualitative research
Keywords:	Aging, Nursing Care, Patients, Primary Prevention, QUALITATIVE RESEARCH

SCHOLARONE™
Manuscripts

I, the Submitting Author has the right to grant and does grant on behalf of all authors of the Work (as defined in the below author licence), an exclusive licence and/or a non-exclusive licence for contributions from authors who are: i) UK Crown employees; ii) where BMJ has agreed a CC-BY licence shall apply, and/or iii) in accordance with the terms applicable for US Federal Government officers or employees acting as part of their official duties; on a worldwide, perpetual, irrevocable, royalty-free basis to BMJ Publishing Group Ltd ("BMJ") its licensees and where the relevant Journal is co-owned by BMJ to the co-owners of the Journal, to publish the Work in this journal and any other BMJ products and to exploit all rights, as set out in our licence.

The Submitting Author accepts and understands that any supply made under these terms is made by BMJ to the Submitting Author unless you are acting as an employee on behalf of your employer or a postgraduate student of an affiliated institution which is paying any applicable article publishing charge ("APC") for Open Access articles. Where the Submitting Author wishes to make the Work available on an Open Access basis (and intends to pay the relevant APC), the terms of reuse of such Open Access shall be governed by a Creative Commons licence – details of these licences and which Creative Commons licence will apply to this Work are set out in our licence referred to above.

Other than as permitted in any relevant BMJ Author's Self Archiving Policies, I confirm this Work has not been accepted for publication elsewhere, is not being considered for publication elsewhere and does not duplicate material already published. I confirm all authors consent to publication of this Work and authorise the granting of this licence.

For peer review only

Barriers and facilitators to pressure ulcer prevention behaviours by older people living in their own homes and their lay carers: A qualitative study

Objective: To identify barriers and facilitators to pressure ulcer (PU) prevention behaviours in community dwelling older people and their lay carers.

Design: Theoretically informed qualitative interviews with two phase, deductive then inductive thematic analysis.

Setting: The study was conducted in one geographical region in the United Kingdom, spanning several community NHS Trusts.

Participants: Community dwelling older patients at risk of pressure ulcer development (n=10) and their lay carers (n=10).

Results: Six themes and subthemes were identified: i) knowledge and beliefs about consequences (nature, source, timing and taboo), ii) social and professional role and influences (who does what, conflicting advice and disagreements), iii) motivation and priorities (competing self-care needs and carer physical ability), iv) memory, v) emotion (carer exhaustion and isolation, carer role versus partner role and patient feelings) and vi) environment (human resource and response, and equipment).

Conclusions: There is minimal research in pressure ulcer prevention in community dwelling older people. This study has robustly applied the theoretical domains framework to understanding barriers and facilitators to pressure ulcer prevention behaviours. Our findings will support co-design of strategies to promote preventative behaviours and are likely to be transferable to comparable health care systems nationally and internationally.

Strengths and limitations

- A structured theoretical approach was adopted to data collection and analysis.
- Both inductive and deductive understandings of barriers and facilitators to PU prevention behaviours were gained, leading to a detailed analysis of influencing factors.
- Service users were involved in the design, analysis and reporting of this research.
- Recruitment was undertaken in a single geographical area.

For peer review only - http://bmjopen.bmj.com/site/about/guidelines.xhtml

For peer review only

Barriers and facilitators to pressure ulcer prevention behaviours by older people living in their own homes and their lay carers: A qualitative study

Introduction

Pressure ulcers (PUs) are localised injuries to the skin and underlying tissues, usually occurring over a bony prominence, resulting from pressure, or pressure combined with shear (1). Globally, a PU prevalence of 12.8% and 11.6% has been reported among hospitalised patients (2) and nursing home residents (3), respectively. Pressure ulcers lead to an increased risk of hospital admission, and treatment is costly, placing a substantial burden on care providers (4). PUs have a detrimental impact on the quality of life of affected individuals (5,6) and are associated with increased morbidity, mortality, pain, fear and despondency (6,7). Whilst international guidelines recommend PU prevention behaviours, these focus on a hospital population and there is a dearth of research surrounding transferability to community settings (8). A Priority Setting Partnership involving 500 patients, lay carers and healthcare practitioners (HCP) identified *Understanding the impact of patient and lay carer involvement* as the second most important priority for PU prevention (9).

Existing evidence-based guidelines offer staff intensive PU prevention strategies including i) repositioning at least every four to six hours, ii) use of appropriate devices (e.g., pressure-relieving mattresses), iii) frequent skin inspection and skin care, and iv) optimal nutrition and hydration (10,11). Although these strategies are appropriate in all settings, home care has unique challenges that limit their applicability. Nursing staff and paid carers are able to spend only limited time with patients, so engagement of patients and lay carers in PU prevention is essential. However, there is limited empirical evidence regarding adherence of patients and lay carers to PU prevention guidance. To date, interventions to enhance patient and lay carer PU prevention behaviours have focused on education, though these have had uncertain impact on knowledge or the number of new pressure ulcers (11). This suggests knowledge might not be the only factor for non-adherence (12).

When developing strategies or interventions to support health behaviour change, explicit use of theory allows an understanding of behavioural determinants (barriers and facilitators), thus enabling mapping of empirically tested behaviour change techniques (13,14). The Theoretical Domains Framework (TDF) (15) synthesises all published theories of behaviour or behaviour change into 11 accessible theoretical domains. These domains offer a comprehensive framework of all potential determinants of behaviour (*knowledge, skills, social/professional role and identity, beliefs about capabilities, beliefs about consequences, motivation and goals, memory attention and decision processes, environmental context and resources, social influences, emotion and action planning*).

For peer review only - http://bmjopen.bmj.com/site/about/guidelines.xhtml

For peer review only

Identifying key determinants using the TDF supports identification of the behaviour change techniques (BCTs) most likely to be effective (16).

Objective

To identify barriers and facilitators to PU prevention behaviours in community dwelling older people and their lay carers.

Methods

Design

Qualitative interviews were conducted to elicit barriers and facilitators to PU prevention behaviours. The interview schedule was informed by domains of the TDF.

Participants

Participants were older adults and lay carers. Older adults were community dwelling in their own home, aged ≥65 years, receiving community healthcare and assessed as being at risk of developing a PU (irrespective of current and previous PU status). Lay carers were those providing unpaid care (for people fitting our criteria for older adult participation), of any kind (e.g., physical, household or other practical support). Exclusion criteria included lacking capacity (patients and carers), patients who had not been assessed for pressure ulcer risk, and carers supporting someone who did not fulfil the patient inclusion criteria. We aimed to recruit a minimum of ten patients and ten carers (n=20) which we expected to be sufficient to achieve data saturation given the theoretical underpinning of the study (17).

Patient and public involvement

In this study we had the input of two patient and public involvement colleagues. One contributed to the study design and the other to design, interpretation of data, reading and commenting on results and writing up. We worked closely with the local Carers Centre who advised on and supported recruitment and hosted a dissemination event for the local community.

Recruitment

Patient participants were recruited through HCPs at a community NHS Trust who provided written information about the study. Several patients declined to take part as they felt that the study was not relevant to their circumstances. Carers were recruited through leaflets distributed through a range of outreach events and community settings, for example, carers centre, churches, community centres, voluntary organisations and supermarkets. Snowballing techniques were used to enhance recruitment. Recruitment of carers began in November 2021 and of patients in March 2022, and

For peer review only - http://bmjopen.bmj.com/site/about/guidelines.xhtml

continued until May 2023. Potential participants were provided with information about the project, including the reasons for the study. Interested individuals were contacted by the research team approximately a week later, to discuss the study and decide if they wished to participate.

Data collection

Written consent was gained at the start of the interview. Interviews took place in a venue of the participants choice (usually their own home). Interviews were undertaken by female, post-doctoral researchers (CS, MW or JR) who were not known to participants prior to being recruited into the research, and who introduced themselves and their backgrounds at the start of the interviews. Participants were offered a gift voucher or BACS payment post-interview. Single interviews were audio recorded and transcribed verbatim. Throughout most interviews, only the researcher and participant were present, though on occasion the person cared for, or the carer were present. One interview was undertaken jointly with the patient and their carer. Notes were taken by the researcher as prompts, these did not form part of the analysis. Transcripts were not returned to participants for comment due to the potentially onerous nature of such checking, particularly with this group for whom it could be considered unethical given the time commitments of carers in particular.

Data analysis

Transcripts were imported into NVivo (v12) and underwent a two-stage analysis. After deductively categorising data to the domains of the TDF, inductive thematic analysis was undertaken according to a six-step process (familiarisation, code generation, combining codes to themes, reviewing themes, determining theme significance and reporting) (18). Data analysis and collection were iterative processes and continued until saturation was achieved. Analysis was initially undertaken by JD, MW and JR and reviewed by the author team.

Results

Characteristics of participants

Table 1 presents participant characteristics (n=20). Patient participants (women n=4, men n=6) were aged 68 to 94 years (mean age 79.5). Three had a current PU, four had previously had PU(s), two had never had a PU and one currently had and had previously experienced PUs. Where patient participants had experience of a PU, five had developed these in hospital and three at home. All patient participants had limited mobility. Lay carer participants (women n=7, men n=3) supported or had supported older relatives, 80% of these participants cared for someone who had a PU. Ages

For peer review only

ranged from 56 to 82 years (mean 75.0). Eight lay carers were caring for their partner (husband or wife), one was caring for their mother-in-law and father-in-law, and one for their mother.

For peer review only

For peer review only - http://bmjopen.bmj.com/site/about/guidelines.xhtml

Table 1: Participant Characteristics[1]

Pseudonym	Role	Gender	Health and self-reported mobility status of patient	Self-reported health status of lay carer
Pt1	Patient	Male	Lower body paralysis, wheelchair to mobilise	
Pt2	Patient	Female	Multiple long-term conditions (LTCs), poor mobility, mobilises using a walking frame	
Pt3	Patient	Male	Paralysis of legs, wheelchair to mobilise	
Pt4	Patient	Male	Usually mobile, currently compromised mobility post-surgery	
Pt5	Patient	Female	Limited mobility using aids (walking frame, stairlift, rails, mobility scooter)	
Pt6[1]	Patient	Male	Multiple LTCs, usually mobile with a recent period of immobility	
Pt7[2]	Patient	Female	Walks short distances and uses a wheelchair	
Pt8	Patient	Male	Multiple LTCs, walks at home with frame and two people, otherwise wheelchair user	
Pt9[3]	Patient	Male	Paraplegic, wheelchair user	
Pt10[4]	Patient	Female	Multiple LTCs, wheelchair user	
Ca1	Wife caring for husband (now deceased)	Female		Osteoporosis
Ca2[3]	Wife caring for husband	Female		Good physical health

[1] Where patient and carer were related this is indicated by superscript matching numbers next to participant pseudonym.

For peer review only - http://bmjopen.bmj.com/site/about/guidelines.xhtml

Pseudonym	Role	Gender	Health and self-reported mobility status of patient	Self-reported health status of lay carer
Ca3	Wife caring for husband	Female		Good physical health
Ca4[2]	Husband caring for wife	Male		Good physical health
Ca5[1]	Wife caring for husband	Female		Severe arthritis
Ca6[4]	Husband caring for wife	Male		Good physical health
Ca7	Daughter-in-law caring for mother and father-in-law (now in care home)	Female		Good physical health
Ca8	Son caring for mother	Male		Good physical health
Ca9	Wife caring for husband	Female		Arthritis
Ca10	Wife caring for husband	Female		Good physical health

For peer review only - http://bmjopen.bmj.com/site/about/guidelines.xhtml

Findings

Interviews took a mean of 50 minutes. Six themes (domains of the TDF) and 10 sub-themes were identified (figure 1). Few differences were identified between the barriers and facilitators to PU prevention experienced by carers when compared to those experienced by patients.

Figure 1

Theme 1: Knowledge and beliefs about consequences

Participants generally knew about the strategies to prevent PUs, except for good hydration and nutrition. This knowledge was generated from multiple sources however, in most cases was acquired *after* PU development. The taboo nature of the subject hindered *pre*-PU awareness.

Nature of knowledge

Some found the term pressure ulcer unfamiliar, for example *"it's naivety in that I thought pressure sores were bed sores, so, I thought you only got them in bed"* (Pt9). The three PU prevention strategies known to participants included repositioning and risk of friction when moving, for example, Ca5 stated, *"caused by people spending too much time in one position . . . it limits the blood supply to the skin in that area"*. Many were aware of vulnerable areas particularly referring to the "bottom" with some mentioning back, hips, heels and elbows. Devices were mentioned by most and many used aids to enhance mobility such as frames and 'grab rails' (a bar attached to a wall, usually next to a bath or toilet, which the individual can hold to help them get up or down). Patient and lay carer participants reported inspecting at-risk areas of the skin and raising concerns with HCPs. Although some participants appreciated the value of good hydration and nutrition, this was rarely linked to prevention of PUs.

Source, timing and taboo

The main source of knowledge for participants was HCPs (in hospital or at home), sometimes because they gave advice but often because the participant observed what they did and adopted these behaviours. Pete (patient) told us *"I've never received any information . . . it's really information I overhear from things like nurses . . . I've looked it up on the internet ."* Some participants had received leaflets from HCPs but found them of limited value, for example, "*if you get a wad of paper the feeling immediately is to dismiss it"* (Pt1). Other knowledge sources included Carers' Centre courses and support groups. The words of Ca1 captured the general consensus *"there*

For peer review only - http://bmjopen.bmj.com/site/about/guidelines.xhtml

definitely needs to be more information out there" and many highlighted their lack of ability to access online information.

Participants learned about PUs *after* they or their relative had developed one. For example, *"the ward did initially* [told me about PUs], *. . . this is what we call a pressure ulcer . . .* [before that] *I knew nothing about them"* (Ca10). Information received in advance of pressure ulcer development was generally not remembered. Another hinderance to PU knowledge was that they were considered unsuitable or too sensitive for discussion (a "no-go" area), with comments such as "*it's one of those things I suppose that when you're sitting in the pub having a quiet pint with someone you don't discuss, do you?"* (Pt4).

Theme 2: Social and professional role and influences

This theme included barriers relating to participants being confused as to the role of visiting practitioners, advice which appeared to conflict and disagreements between practitioners or practitioners and carers.

Who does what?

Most participants were unclear about what practitioners did, the purpose of their visit and who to approach about PUs. Supplementary Table 1 illustrates the nature and number of health and social carers with whom individuals were in contact over the previous year. In extreme cases the volume and number of visits left participants reflecting a sense of chaos, for example, Ca1, *"four people coming a day, nurses, carers, doctors . . . physios somebody knocking on your door, it's hard . . I've had the physio', occupation therapist . . . they bring the tissue viability nurse and some other ladies . . . two ladies, um, I think they must be physios I think"*. Similarly, Ca10 said *"there's an older lady who's very good and she just gets on . . . does whatever is needed. And then this other person will suddenly turn up and you think, why are you here?"*.

Supplementary table 1

For peer review only - http://bmjopen.bmj.com/site/about/guidelines.xhtml

Conflicting advice and disagreements

Participants described variations in practitioner advice and being asked to pass instructions from one to another, causing distress and awkwardness. Ca3 explained *"we're in the middle, we've got a podiatrist telling us . . . 'will you tell your nurses about it?' Then we've had to relay that to three or four different nurses who see* [husband]*".* Sometimes people reported conflicts between what the professional suggested and what they, their family members or lay carers thought best. Pt3 described how his live-in carer reacted when he did not agree with the physiotherapist's advice, *"nearly took her head off . . . he gets a bit agitated, my carer".* On occasion, lay carers' advice took priority over practitioners', *"I take all the advice that* [wife] *ever gives . . . but in general I have faith in the NHS*" (Pt6). Ca4 described an ongoing battle with district nurses regarding pressure relieving equipment, *"she had oedema . . . incredibly wet and sweaty in the plastic, which I didn't think was a very good idea . . . I kept taking them off and the nurses kept telling me off and putting them back on again".* Sometimes participants were concerned disagreements would negatively impact care, *"you've got to be careful because you don't want to upset these people who are caring for* [husband]" (Ca3). Similarly, patients and lay carers did not always agree on care, in these instances the burden of the disagreement seemed more emotionally arduous for both parties. Some carers described how the person cared for found accepting care and adhering to PU prevention behaviours a challenge, "*won't take on board the seriousness of it on and read the information. . .* [he] *would say you're keeping on too much . . . nagging"* (Ca1).

Theme 3: Motivation and priorities

Most patients and carers considered PU prevention to be important but there were two key barriers, patients having to prioritise other physical health needs, and the physical restrictions of lay carers.

Competing self-care needs

This theme related in the most part to repositioning. Nearly all participants reported that the patient was physically unable to adopt PU advice due to physical restrictions. Most often cited were breathlessness, pain or fear of falling. *"I tend not to lie flat . . . I do turn on my side, but not fully"* (Pt6). Pt1 said, *"sometimes, I'm in so much pain in my legs I can't do anything"*. Inspecting vulnerable areas for deterioration was challenging due to physical restrictions, Ca1, speaking about her own vulnerability to PUs found a way around this, she said "*can't see behind . . . I asked my daughter to take a picture".* To a lesser extent, poor health resulted in a reduced appetite, which inhibited good nutrition as a prevention behaviour.

Carer physical ability

For peer review only - http://bmjopen.bmj.com/site/about/guidelines.xhtml

Whilst motivated to provide the best possible care for the person they cared for, some carers were restricted by, and thus had to prioritise, their own health issues, "*I have to hoist him to reposition him every time . . . I am very cautious how I move him now because, you know, I was 51 when I started and I'm now 73"* (Ca2).

Theme 4: Memory

Five people cited memory as a barrier, Ca5 said, *"he needs to be reminded* [to eat and drink]*"* and Ca7, *"needed to be encouraged and reminded* [to change position], *she would forget".* Two participants suggested alarms as reminders, for example *"setting it up on my phone, a reminder"* (Ca4).

Theme 5: Emotion

The emotional burden, including exhaustion and isolation, of giving or receiving care was significant irrespective of the presence of PUs or the need for PU prevention. Some participants spoke of conflicting carer versus partner roles and a range of other feelings.

Carer exhaustion and isolation

Carer exhaustion and isolation was indirectly linked with PU prevention and was a problem expressed by all carers and most patients. Carers felt frustrated, alone and in need of practical and emotional support. "*Carers . . . they're left in a hole on their own and all the time they're trying to get out of this hole and deal with everything . . . it takes a lot of emotional and, and, energy"* (Ca1). Ca5 was reluctant to speak to or seek support from her grown up children, *"I would hesitate to load too much onto them . . . because they care so much about* [husband]*".* Sometimes carers described a mutual "two-way" caring relationship, where support was exchanged, but this was not always the case. Spending time away from the home helped carers to see friends or attend support groups, sometimes making use of "sitters" (a form of respite care where someone from a care company or other organisation comes to the home and spends time with the person being cared for). *"I had a sitting service . . . twice a week . . . a lifesaver. . . it doesn't have to be that you go out"* (Ca1). Social contact for some was nearly impossible, as Ca9 told us, *"if we went somewhere socially, there's not always a disabled toilet and there is no grabrail . . . I miss people".* Emotions impacted on interactions, leading to feelings of guilt, "*there have been* [times] *I have lost the plot . . . I feel awful . . . I have a flare up . . . he says he understands, and he does"* (Ca3). Lay carers sometimes found professional care visits an intrusion of privacy and closeness, *"my house was no longer my home, I lost that. And I think we lost a little bit of our closeness"* (Ca2). Beyond exhaustion and isolation, carers listed many emotions, for example, fear of their partner dying before them, or of their partner being left behind should they die, guilt that their care was insufficient or inadequate, worries about

For peer review only - http://bmjopen.bmj.com/site/about/guidelines.xhtml

finances, frustrations of trying to get additional health or social care help, the vulnerability of needing to rely on others and distress at seeing a relative in pain or unwell.

Caregiver role conflict

Partner carers described personal role conflict and expressed differing opinions about the extent of their carer role. For example, *"I personally didn't help* [PU care], *no, it came through the nurses"* (Ca1), in contrast another carer explained *"I don't particularly want anyone to look after him . . . intrusion of our closeness"* (Ca2). Being both partner and carer was a concern. Often this was about dignity and not allowing the "partnership" to be subsumed by a caring role. Carers dealt with this by compartmentalising. For example, Ca1, *"we have a plan, my husband and I, it's like I'm always going to be his wife, and when I do certain things . . .I say to him, I've got my carer's hat on . . . things that aren't quite so nice . . . I feel I'm two different people".*

Patient feelings

Patients reported malaise, low energy and low mood adding to the challenge of PU prevention. *"The body doesn't last forever . . . it is frustrating. . . can't even do the simplest things . . .life isn't a bed of roses when you get older"* (Pt1). Carers recognised and empathised, for example, "[it's] *sad, this fiercely independent, highly intelligent man is just not able to do things anymore"* (Ca5). Patients were embarrassed by younger formal carers inspecting parts of their body, feelings carers empathised with, *"I mean we laugh and joke about it . . that was my way of dealing with . . . it's embarrassment"* (Pt4) and *"I always did what I call his private bits . . . washed and creamed him . . . some of the girls are 17 and 18 years old . . . it's his dignity"* (Ca9).

Theme 6: Environment

The key environmental barrier was human resources. Equipment was generally available but at times provided too late.

Human resource shortage and heavy burden of care

Over half of participants spoke about the frustrations of getting help from HCPs or making appointments. "[I] *want to scream . . . you spend half your life on the telephone trying to chase things . . . it takes a lot of emotional . . . and energy . . . nurses not turning up . . . transport doesn't turn up . . . that happens such a lot"* (Ca1). Out of hours provision was a problem, *"I dread every bank holiday . . . if something is going to go wrong"* (Ca2). Participants believed that practitioners were short of time, short of funds, overworked or stressed, *"overburdened . . . I know they are just overworked, spread too thin"* (Ca7).

Equipment

For peer review only - http://bmjopen.bmj.com/site/about/guidelines.xhtml

Equipment considered necessary by HCPs caring for patient participants was provided, including profiling beds, bed extensions, walking frames, hoists, cushions, offloading wedges, mattresses and handrails. The only concerns were lack of support to install it and delays in receiving it. For example, Pt2 remembered, *"this man, he said mattress, and he plonked the tubes down. So, I said, well what do I do with them? He said, haven't you got anyone here? I said no. Oh, I'll do it, he said . . . the bloody fool . . . he'd just left it on top* [of bedding]*"*.

Discussion

In interviews underpinned by the TDF (15) with ten patients and ten carers we identified six themes representing barriers to PU prevention behaviours: i) knowledge and beliefs about consequences, ii) social or professional role and influences, iii) motivation and priorities, iv) memory, v) emotion and vi) environment.

Although most barriers identified were individual in nature, one significant barrier, the late acquisition of PU knowledge (*after* PU development), partly attributable to its taboo nature, suggests population level strategies are needed. In cases of similarly taboo areas of health, such as bowel cancer, campaigns have achieved some success. A recent campaign, involving diary style podcasts, led by a young woman with terminal bowel cancer, destigmatised "toilet habits" and led to a tenfold increase in online searches for bowel cancer symptoms immediately following her death (19). For prostate cancer, there was a tenfold increase in use of a publicly available "30-second checker" prostate screening tool after a television presenter announced his diagnosis (20). Social media and online campaigns are not accessed by or useful to all (21). For the populations in question, predominantly older people, this may offer only a partial solution. For example, systematic review evidence reports that 67% of older people access the internet compared with 90% of all adults (22). In addition, recent research shows that internet campaigns are less effective for men than women (22). We were unable to identify any research evaluating the outcome of the annual European Pressure Ulcer Advisory Panel (EPUAP) 'Stop Pressure Ulcers' campaign designed to raise awareness of PUs. However, such campaigns may have a value in that those accessing may influence others (e.g., women and younger people influencing male and older relatives) and indeed the younger people of today are our future "at risk" generation.

When seeking to address individual barriers there is a need for solutions to be tailored to the individual (23). The TDF can be used to identify the specific behaviour change techniques (BCTs) that are most likely to be effective (16). This approach has been used and systematically reviewed in the case of both HCPs (24) and patients and carers (25). These BCTs can be translated into pragmatic intervention components. For example, where knowledge is lacking, information may be the BCT of

For peer review only - http://bmjopen.bmj.com/site/about/guidelines.xhtml

choice, where motivation is in deficit, BCTs such as goal setting, rewards and consideration of pros and cons to the behaviour in question may offer support (16). A co-design approach involving end users (26) is most likely to result in a product that is acceptable, practicable and equitable (27). There is published precedent (27), and systematic review guidance (28), for high quality design with older people. Elements of co-design are used in the United Kingdom the National Wound Care Strategy Programme in which the Patient Experience Network have created resources by patients for patients. However, to date focus has been on wound care rather than specifically pressure ulcers.

Strengths and limitations

There were strengths and limitations to our study. We used a structured theoretical approach to data collection and analysis. Service users were involved in design, analysis and reporting. Most research relating to PUs considers hospital settings and health care practice. To the best of our knowledge this is the first to consider community dwelling older patients and their lay carers. We recruited from only one geographical area, although our results may be transferrable it is possible that elements of service provision may differ both nationally and internationally. Whilst we sought to establish facilitators (as well as barriers) these related only to the provision of equipment and were therefore not helpful in informing future solutions.

HCPs providing care to community dwelling older people need to give timely and consistent PU prevention messages, in an accessible format to patients and lay carers. Greater clarity in roles and responsibility among care providers and across HCP and lay carer boundaries would be valuable.

As our research is, to the best of our knowledge, the first to investigate barriers and facilitators to PU prevention in community dwelling older people, further research is needed. Now we understand the barriers there is an opportunity to co-design strategies to support PU prevention behaviours in older people and test these in practice.

Conclusion

Little research has considered the contribution made by community-dwelling patients and their lay carers in preventing pressure ulcers. This study sought to understand the barriers and facilitators to engaging in pressure ulcer prevention through robust application of the theoretical domains framework. Our findings demonstrate that the key barriers relate to knowledge and beliefs about consequences, social and professional roles and influences, motivation and priorities, memory, emotion, and environment. This knowledge will underpin further research required to co-design strategies to promote preventative behaviours around pressure ulcer prevention for this population.

For peer review only - http://bmjopen.bmj.com/site/about/guidelines.xhtml

For peer review only

Recommendations for clinical practice include ensuring that patients and their lay carers are provided with consistent, early information about PU prevention strategies and that carers are offered support. The learning from this project is likely to be transferable to comparable national and international health care systems.

Funding statement

This project is funded by the National Institute for Health and Care Research (NIHR) under its Research for Patient Benefit (RfPB) Programme (Grant Reference Number NIHR201933). The views expressed are those of the author(s) and not necessarily those of the NIHR or the Department of Health and Social Care.

Competing interests

None declared

Authors contributions

JR, MW, FC, JD, AD and KO designed the study. JR and MW contributed to data collection. JD, MW and JR contributed to data analysis. FC, JD, JR and MW drafted the manuscript. MW, JR, KO and AD critically revised the manuscript. JR is responsible for the overall content as the guarantor, had full access to the data and takes responsibility for the integrity of the data and the accuracy of the data analysis.

Acknowledgements

Dr Carol Samuel collected some of the data for this project and began the data analysis.

Data sharing statement

Data are available upon reasonable request, from the corresponding author.

Word count

4266 (not including figures and tables)

Ethics

This study was reviewed and approved by Cornwall and Plymouth Research Ethics Committee (reference 21/SW/0061).

For peer review only

References

1. European Pressure Ulcer Advisory Panel, National Pressure Ulcer Advisory Panel, Pan Pacific Pressure Injury Alliance (2019) Prevention and treatment of pressure ulcers/ injuries: Clinical Practice Guideline. The International Guideline.
2. Li Z, Lin F, Thalib L, et al. Global prevalence and incidence of pressure injuries in hospitalised adult patients: A systematic review and meta-analysis. *International journal of nursing studies* 2020;105:103546.
3. Sugathapala RUP, Latimer S, Balasuriya A, et al. Prevalence and incidence of pressure injuries among older people living in nursing homes: A systematic review and Meta-analysis. *International Journal of Nursing Studies* 2023:104605.
4. NHS Digital. Hospital Admitted Patient Care Activity 2017-2018 [Available from: https://digital.nhs.uk/data-and-information/publications/statistical/hospital-admitted-patient-care-activity/2018-192023.
5. Gorecki C, Brown JM, Nelson EA, et al. Impact of pressure ulcers on quality of life in older patients: a systematic review. *Journal of the American Geriatrics Society* 2009;57(7):1175-83.
6. Jackson DE, Durrant LA, Hutchinson M, et al. Living with multiple losses: Insights from patients living with pressure injury. *Collegian* 2018;25(4):409-14.
7. Landi F, Onder G, Russo A, et al. Pressure ulcer and mortality in frail elderly people living in community. *Archives of gerontology and Geriatrics* 2007;44:217-23.
8. Roddis JK, Liversedge HL, Ryder I, et al. Incorporating the patient experience into clinical guidelines: recommendations for researchers and guideline developers. *BMJ Evidence-Based Medicine* 2019;24(4):125-26.
9. James Lind Alliance Priority Setting Partnership. Pressure Ulcers Top 10 [Available from: https://www.jla.nihr.ac.uk/priority-setting-partnerships/pressure-ulcers/top-10-priorities/2023.
10. National Institute for Health and Care Excellence. Pressure ulcers: prevention and management Clinical guideline 2014 [Available from: www.nice.org.uk/guidance/cg1792023.
11. O'Connor T, Moore ZE, Patton D. Patient and lay carer education for preventing pressure ulceration in at-risk populations. *Cochrane Database of Systematic Reviews* 2021(2)
12. MCCOULOUGH S. Implementing an adapted SSKIN bundle and visual aid in the community setting. *Wounds UK* 2016;12(1)
13. Skivington K, Matthews L, Simpson SA, et al. A new framework for developing and evaluating complex interventions: update of Medical Research Council guidance. *bmj* 2021;374
14. National Institute for Health and Care Excellence, Behaviour change: individual approaches Public health guideline [PH49] 2014 [Available from: https://www.nice.org.uk/Guidance/PH49.
15. Michie S, Johnston M, Abraham C, et al. Making psychological theory useful for implementing evidence based practice: a consensus approach. *BMJ Quality & Safety* 2005;14(1):26-33.
16. Michie S, Johnston M, Francis J, et al. From theory to intervention: mapping theoretically derived behavioural determinants to behaviour change techniques. *Applied psychology* 2008;57(4):660-80.
17. Francis JJ, Johnston M, Robertson C, et al. What is an adequate sample size? Operationalising data saturation for theory-based interview studies. *Psychology and health* 2010;25(10):1229-45.
18. Braun V, Clarke V. Using thematic analysis in psychology. *Qualitative research in psychology* 2006;3(2):77-101.
19. NHS England. Lasting gifts of "bowel babe" Dame Deborah continue in East of England 2023 [Available from: https://www.england.nhs.uk/east-of-england/2023/06/28/2023.
20. BBC News. Nick Owen: Prostate cancer charity sees increase in demand 2023 [Available from: https://www.bbc.co.uk/news/uk-england-birmingham-664391222023.

For peer review only - http://bmjopen.bmj.com/site/about/guidelines.xhtml

21. Patel MS, Halpern JA, Desai AS, et al. Success of prostate and testicular cancer awareness campaigns compared to breast cancer awareness month according to internet search volumes: a google trends analysis. *Urology* 2020;139:64-70.
22. Hunsaker A, Hargittai E. A review of Internet use among older adults. *New media & society* 2018;20(10):3937-54.
23. Baker R, Camosso-Stefinovic J, Gillies C, et al. Tailored interventions to address determinants of practice. *Cochrane Database of Systematic Reviews* 2015(4)
24. Dyson J, Cowdell F. How is the Theoretical Domains Framework applied in designing interventions to support healthcare practitioner behaviour change? A systematic review. *International Journal for Quality in Health Care* 2021
25. Cowdell F, Dyson J. How is the Theoretical Domains Framework applied in health behaviour change? A systematic search and narrative synthesis. *PROSPERO International prospective register of systematic reviews* 2018
26. Jackson CL, Greenhalgh T. Co-creation: a new approach to optimising research impact. *Med J Aust* 2015;203(7):283-4.
27. Glover L, Dyson J, Cowdell F, et al. Healthy ageing in a deprived northern UK city: A co-creation study. *Health & Social Care in the Community* 2020;28(6):2233-42.
28. Cowdell F, Dyson J, Sykes M, et al. How and how well have older people been engaged in healthcare intervention design, development or delivery using co-methodologies: A scoping review with narrative summary. *Health & Social Care in the Community* 2020

For peer review only

For peer review only - http://bmjopen.bmj.com/site/about/guidelines.xhtml

Figure Legend:

Figure 1 – Themes and subthemes

For peer review only

For peer review only - http://bmjopen.bmj.com/site/about/guidelines.xhtml

Figure 1: Themes and subthemes

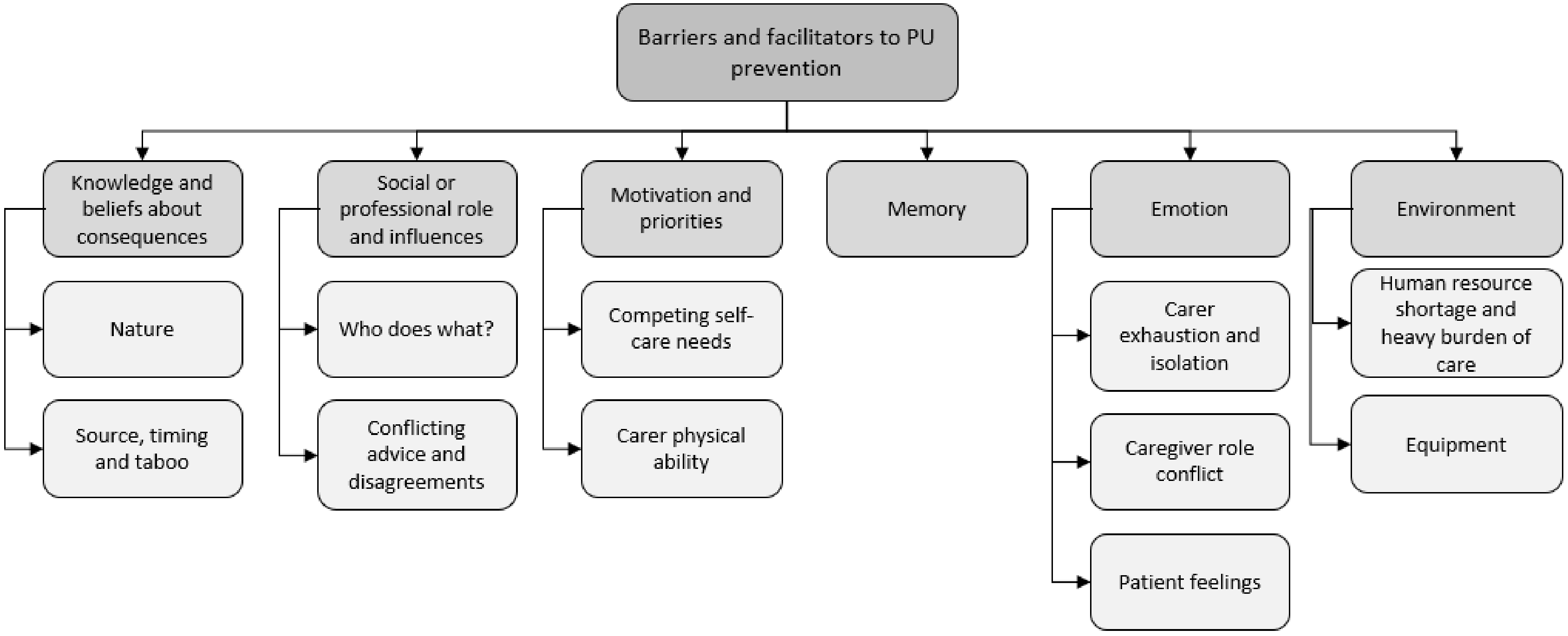

Supplementary Table 1: Community health and social services supporting patients (✔) and carers (*) in the last year

Pseudonym	Role	Carer centre	Day centre	District nurse	Family carer live-in	Family carer visiting	GP	Occupational therapist	Paid carer live-in	Paid carer visiting	Pharmacy	Physiotherapist	Podiatrist	Residential respite	Sitter	Social prescriber	Social worker	Specialist nurse	Speech and language	Tissue viability nurse
Pt1	Patient			✔		✔		✔	✔	✔										
Pt2	Patient			✔		✔		✔			✔	✔								
Pt3	Patient			✔		✔	✔	✔	✔	✔										
Pt4	Patient			✔								✔								
Pt5	Patient			✔	✔															
Pt6	Patient			✔	✔		✔	✔												
Pt7	Patient			✔	✔															
Pt8	Patient			✔		✔	✔	✔		✔	✔	✔	✔			✔				
Pt9	Patient		✔	✔	✔	✔				✔										✔
Pt10	Patient			✔	✔	✔	✔				✔	✔		✔						
Ca1	Carer			✔	✔	✔	✔	✔		✔		✔	✔		*					
Ca2	Carer	*		✔	✔	✔	✔					✔	✔							✔
Ca3	Carer	*			✔			✔		✔			✔							
Ca4	Carer			✔	✔							✔								
Ca5	Carer			✔	✔		✔	✔												
Ca6	Carer				✔	✔	✔				✔	✔		*						
Ca7	Carer			✔	✔	✔		✔		✔				*			✔			
Ca8	Carer	*		✔	✔		✔	✔							✔		✔			
Ca9	Carer			✔		✔	✔	✔		✔					✔			✔	✔	
Ca10	Carer			✔		✔		✔		✔		✔		*						

For peer review only

COREQ (COnsolidated criteria for REporting Qualitative research) Checklist

A checklist of items that should be included in reports of qualitative research. You must report the page number in your manuscript where you consider each of the items listed in this checklist. If you have not included this information, either revise your manuscript accordingly before submitting or note N/A.

Topic	Item No.	Guide Questions/Description	Reported on Page No.
**Domain 1: Research team and reflexivity**			
Personal characteristics			
Interviewer/facilitator	1	Which author/s conducted the interview or focus group?	4
Credentials	2	What were the researcher's credentials? E.g. PhD, MD	4
Occupation	3	What was their occupation at the time of the study?	4
Gender	4	Was the researcher male or female?	4
Experience and training	5	What experience or training did the researcher have?	4
Relationship with participants			
Relationship established	6	Was a relationship established prior to study commencement?	4
Participant knowledge of the interviewer	7	What did the participants know about the researcher? e.g. personal goals, reasons for doing the research	4
Interviewer characteristics	8	What characteristics were reported about the inter viewer/facilitator? e.g. Bias, assumptions, reasons and interests in the research topic	4
**Domain 2: Study design**			
Theoretical framework			
Methodological orientation and Theory	9	What methodological orientation was stated to underpin the study? e.g. grounded theory, discourse analysis, ethnography, phenomenology, content analysis	3
Participant selection			
Sampling	10	How were participants selected? e.g. purposive, convenience, consecutive, snowball	3
Method of approach	11	How were participants approached? e.g. face-to-face, telephone, mail, email	3
Sample size	12	How many participants were in the study?	4
Non-participation	13	How many people refused to participate or dropped out? Reasons?	3
Setting			
Setting of data collection	14	Where was the data collected? e.g. home, clinic, workplace	4
Presence of non-participants	15	Was anyone else present besides the participants and researchers?	4
Description of sample	16	What are the important characteristics of the sample? e.g. demographic data, date	5-6
Data collection			
Interview guide	17	Were questions, prompts, guides provided by the authors? Was it pilot tested?	3
Repeat interviews	18	Were repeat inter views carried out? If yes, how many?	4
Audio/visual recording	19	Did the research use audio or visual recording to collect the data?	4
Field notes	20	Were field notes made during and/or after the inter view or focus group?	4
Duration	21	What was the duration of the inter views or focus group?	7
Data saturation	22	Was data saturation discussed?	4
Transcripts returned	23	Were transcripts returned to participants for comment and/or	4

For peer review only

Topic	Item No.	Guide Questions/Description	Reported on Page No.
		correction?	
**Domain 3: analysis and findings**			
Data analysis			
Number of data coders	24	How many data coders coded the data?	4
Description of the coding tree	25	Did authors provide a description of the coding tree?	4
Derivation of themes	26	Were themes identified in advance or derived from the data?	4
Software	27	What software, if applicable, was used to manage the data?	4
Participant checking	28	Did participants provide feedback on the findings?	4
Reporting			
Quotations presented	29	Were participant quotations presented to illustrate the themes/findings? Was each quotation identified? e.g. participant number	7-12
Data and findings consistent	30	Was there consistency between the data presented and the findings?	7-12
Clarity of major themes	31	Were major themes clearly presented in the findings?	7-12
Clarity of minor themes	32	Is there a description of diverse cases or discussion of minor themes?	10 (theme 4)

Developed from: Tong A, Sainsbury P, Craig J. Consolidated criteria for reporting qualitative research (COREQ): a 32-item checklist for interviews and focus groups. *International Journal for Quality in Health Care*. 2007. Volume 19, Number 6: pp. 349 – 357

Once you have completed this checklist, please save a copy and upload it as part of your submission. DO NOT include this checklist as part of the main manuscript document. It must be uploaded as a separate file.

For peer review only
